# Total Synthesis of (−)-Cylindricine H

**DOI:** 10.1021/acs.orglett.2c02004

**Published:** 2022-07-18

**Authors:** Miriam Piccichè, Alexandre Pinto, Rosa Griera, Joan Bosch, Mercedes Amat

**Affiliations:** Laboratory of Organic Chemistry, Faculty of Pharmacy and Food Sciences, and Institute of Biomedicine (IBUB), University of Barcelona, 08028-Barcelona, Spain

## Abstract

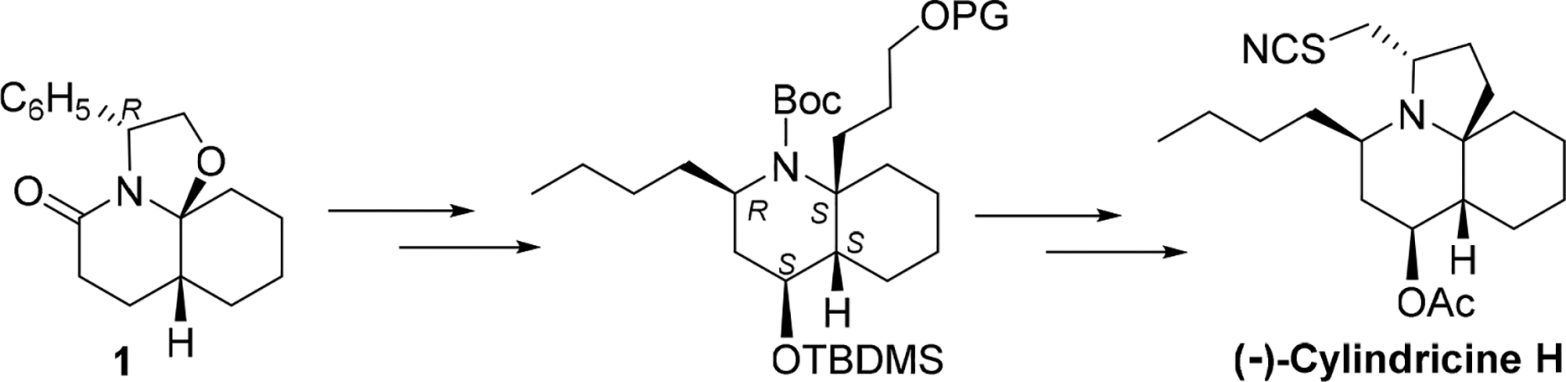

Starting from (*R*)-phenylglycinol-derived tricyclic lactam **1**, the enantioselective synthesis of (−)-cylindricine H is reported. From the stereochemical standpoint, the key steps are the stereoselective generation of the quaternary C_10_ stereocenter, the stereoselective introduction of the C_4_ acetoxy and C_2_ butyl substituents taking advantage of the lactam carbonyl functionality, and the assembly of the pyrrolidine ring with the required functionalized one-carbon chain at C_13_ by intramolecular opening of an epoxide.

Cylindricines are a small group of 11 marine alkaloids isolated in the early 1990s by Blackman et al. from the ascidian *Clavelina Cylindrica* off the coast of Tasmania.^1^ They exhibit a pyrrolo[1,2-*j*]quinoline (cylindricines A, C–I, K) or pyrido[2,1-*j*]quinoline (cylindricines B, J) azatricyclic framework (Figure 1). Cylindricines A and C–G have in common a six-carbon lateral chain (except cylindricine G) and a ketone on the B ring but differ in the functionality of the one-carbon appendage on the pyrrolidine ring. Cylindricine A, with a chloromethyl group, is in equilibrium with its pyridoquinoline congener cylindricine B, presumably via an aziridinium intermediate. Cylindricines H–J, isolated from the same sources, have a butyl instead of a hexyl substituent at C-2 and were the first 4-acetoxycylindricines to be described. Cylindricines I and J bear an isothiocyanate at the one-carbon chain on the pyrrolidine ring, while cylindricines F–H have a thiocyanate and are the only compounds isolated from ascidians with these functionalities. Finally, cylindricine K resembles cylindricine A, but the carbocyclic A ring bears an unsaturated ketone. The optical rotation of these natural products was not determined and, consequently, their absolute configuration remained unassigned.

**Figure 1:**
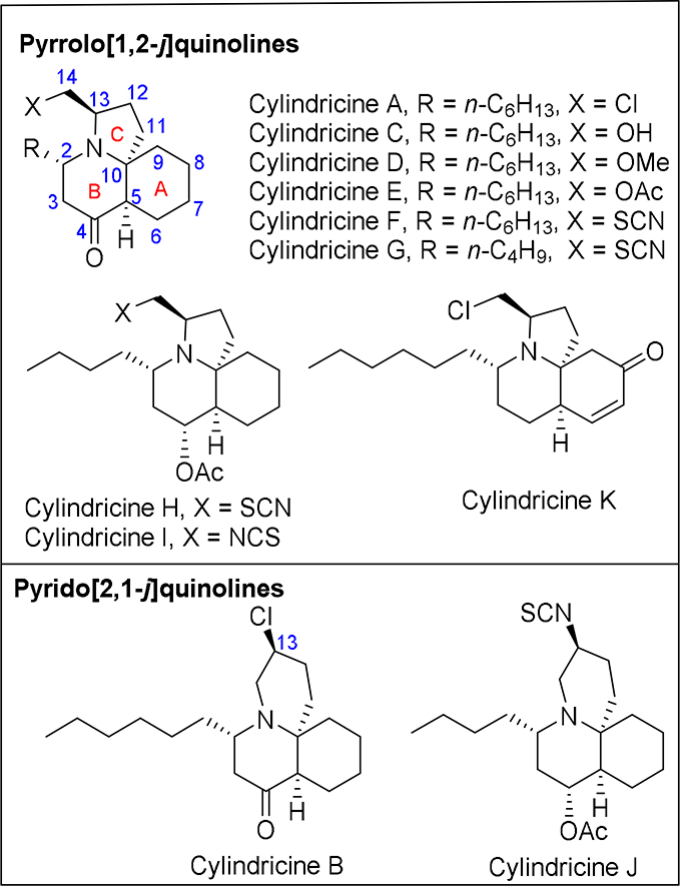
Alkaloids of the cylindricine group.

Since their isolation, owing to their unique structure, cylindricines have attracted significant attention among the synthetic community and currently the total synthesis of racemic cylindricines A–E has been achieved by several authors.^2^ However, all the enantioselective syntheses reported to date focus on cylindricine C, which can be readily converted into cylindricines D and E.^3^ No total synthesis of cylindricines F–K has been described in the literature.

In recent work we have explored the stereoselective generation of chiral aminoalcohol-derived oxazoloquinolone tricyclic lactams and their transformation into diversely substituted *cis*-decahydroquinolines (DHQs). The relevance of these enantiomeric scaffolds in the total synthesis of alkaloids having in common a DHQ nucleus was illustrated with the total synthesis of *Myrioneuron*,^4^*Lycopodium*,^5^ amphibian,^6^ and marine alkaloids.^7^ To further demonstrate the synthetic utility of these chiral tricyclic lactams, we decided to undertake the more challenging total synthesis of cylindricine H, which requires the formation of a quaternary carbon center embedded within a complex azatricyclic system.

With this purpose in mind, (*R*)-phenylglycinol-derived tricyclic lactam **1**,^8^ bearing the DHQ moiety (rings AB of cylindricine H), was envisaged as the starting enantiomeric scaffold (Scheme 1). The synthetic strategy involves the initial generation of the quaternary stereocenter by the introduction of a functionalized carbon chain at the angular C_11a_ position (C_10_ of cylindricine) taking advantage of the *N*-acyl hemiaminal function. The subsequent elaboration of this chain would allow the closure of the C ring in the final steps by intramolecular opening of an epoxide. The remaining acetoxy and butyl substituents would be stereoselectively incorporated by synthetic manipulation of the amide. A β-boration of the corresponding α,β-unsaturated amide, followed by oxidation of the C–B bond and acetylation of the resulting alcohol, would be used to stereoselectively introduce the acetoxy group, whereas activation of the amide, followed by the introduction of the butyl substituent and stereoselective reduction of the resulting enamide via an acyliminium salt, would install the lateral chain of cylindricine H.

**Scheme 1:**
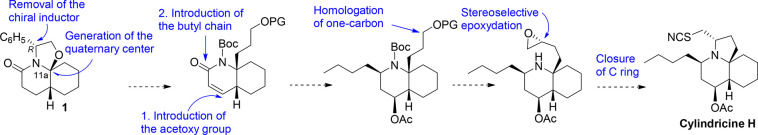
Synthetic Strategy

Initial attempts to introduce an allyl chain at the angular position of tricyclic lactam **1** under the conditions reported by Danishefsky^9^ from the 5–5–5 lactam **2** resulted in failure. The lower angular strain of the 5–6–6 system present in **1** due to the larger size of the B ring could account for this result. In fact, in our hands, under these conditions, the 5–5–6 tricyclic lactam **3** stereoselectively provided the allylated product **4**(10) in 75% yield (Scheme 2). To increase the reactivity of the hemiaminal moiety, the carbonyl of lactam **1** was selectively reduced by conversion into the corresponding thiolactam **5** followed by treatment with NaBH_4_. Then, the quaternary stereocenter was satisfactorily installed by reaction of **6** with allylmagnesium bromide to give the desired allylated compound **7** in excellent stereoselectivity (d.r. 96:4) and good overall yield from **5**. The stereochemical outcome of the reaction was confirmed by conversion of **7** into the corresponding *N*-Cbz *cis*-DHQ **8** (see SI for details), which has been reported in the racemic series.^11^

**Scheme 2:**
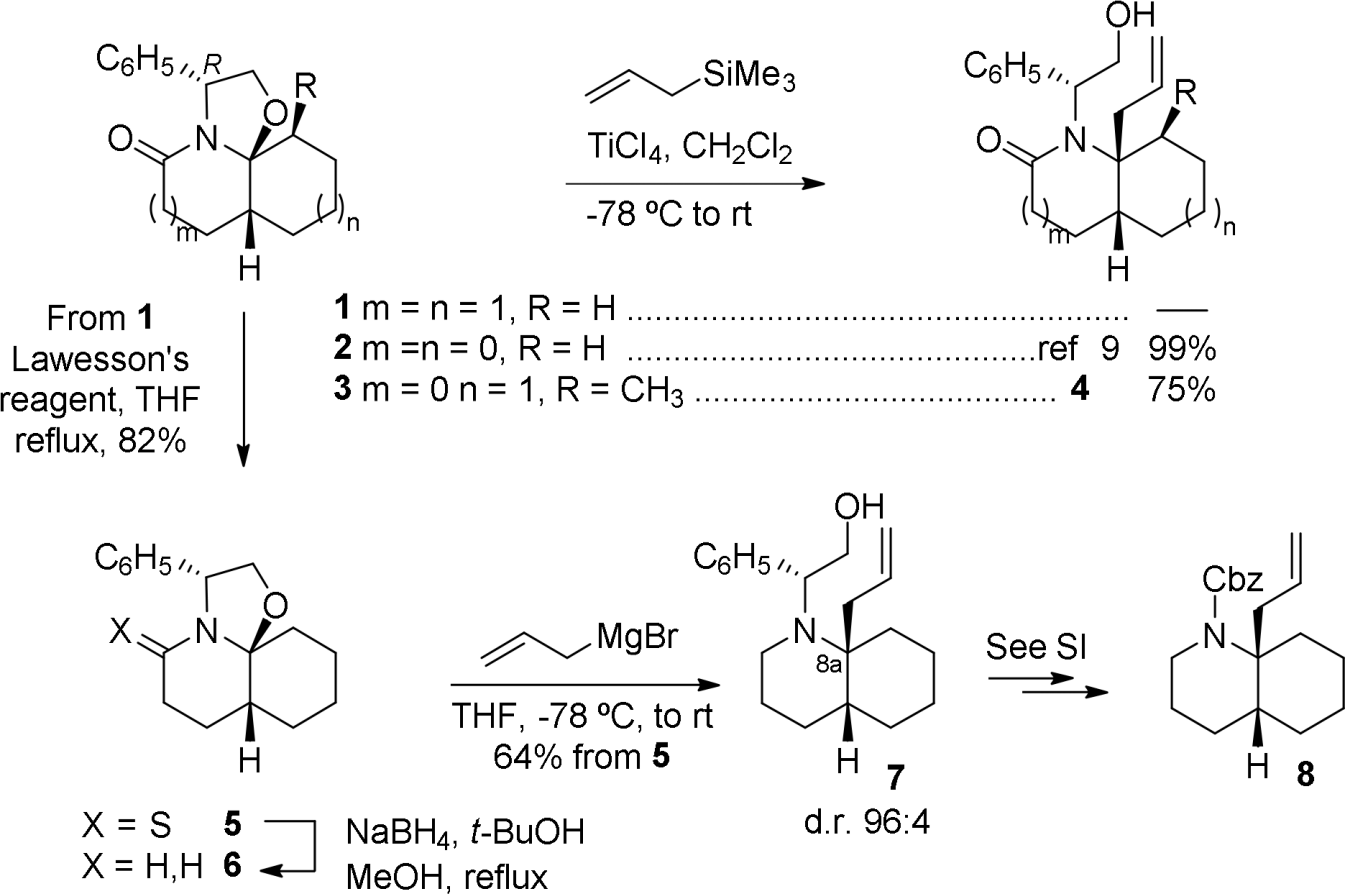
Allylation of Tricyclic Lactams by Amidoalkylation

Our next goal was the removal of the chiral inductor from **7** and the refunctionalization of the C_2_-position by oxidation to the corresponding lactam. Hydroboration–oxidation of the double bond present in **7** afforded diol **9** in excellent yield. The subsequent catalytic hydrogenation of **9** caused *N*-debenzylation, and the resulting amino-alcohol was sequentially protected as *O*-silyl and *N*-Boc derivatives, leading to the C_8a_ substituted *cis*-DHQ **10** in good overall yield. At this point, the lactam carbonyl was efficiently reinstalled by ruthenium-promoted oxidation to give *cis*-DHQ-2-one **11**.

We planned to incorporate the C_4_ oxy substituent of cylindricine H by conjugate addition of a diboron reagent followed by oxidation of the C–B bond. The required unsaturated lactam **12** was prepared by phenylselenation of the lithium enolate of **11** and subsequent oxidation. Copper-catalyzed conjugate addition of bis(pinacolato)diboron to **12**, under the conditions reported by Yun,^12^ and consecutive oxidation with sodium perborate furnished an alcohol, which was protected as a silyl derivative without further purification. Compound **13**, with the appropriate configuration for the synthesis of cylindricine H, was obtained in excellent stereoselectivity and good overall yield (Scheme 3).

**Scheme 3:**
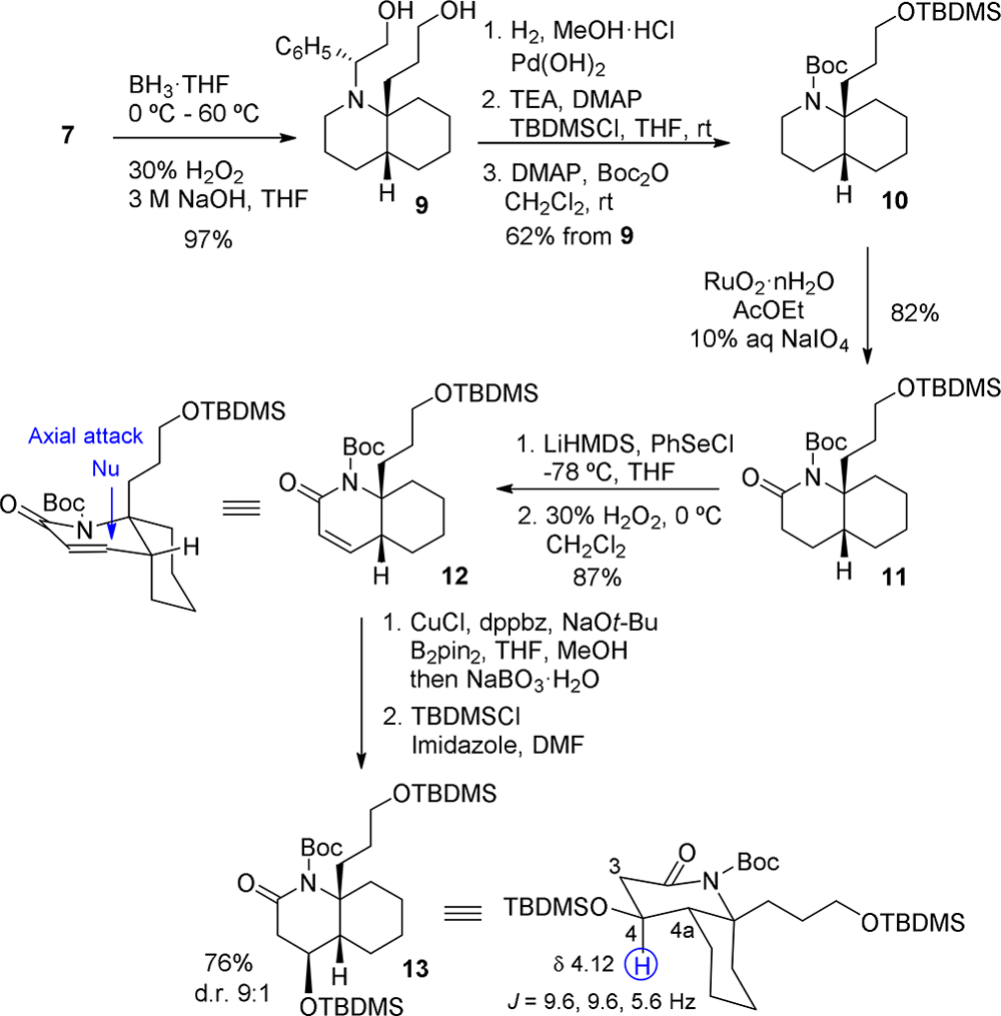
Stereocontrolled Introduction of the C_4_ Substituent

The stereochemical outcome of the β-boration reaction can be rationalized by considering an axial attack of the nucleophile, under stereoelectronic control,^13^ on the more accessible convex face of the unsaturated lactam **12**, which adopts a conformation in which the C_8a_ chain is pseudoaxial with respect to the unsaturated ring to avoid the 1,3-diaxial destabilizing interactions that would appear in the alternative conformation.

For the introduction of the butyl chain, we used a procedure similar to the one we employed in our synthesis of gephyrotoxine 287C,^5b^ which included the generation of an enecarbamate by coupling of a vinyl triflate with an organometallic reagent, followed by protonation and stereoselective reduction of the resulting acyliminium salt. It was assumed that the intermediate acyliminium salt **A** would adopt a conformation in which the OTBDMS and (CH_2_)_3_OTBDMS substituents would be equatorial with respect to the heterocyclic ring. An axial attack of the hydride ion, under stereoelectronic control, would lead to the required 2-butyl-DHQ, with the C_2_ configuration of cylindricine H. Triflate **14** was prepared by treatment of lactam **13** with LiHMDS at −78 °C for short reaction times and subsequent reaction of the lithium enolate with Comins’ reagent at this temperature. The crude mixture was immediately treated with a solution of CuI and *n*-BuLi at low temperature, and the resulting enecarbamate **15** was reduced with NaBH_3_CN–TFA without purification to give trisubstituted DHQ **16** in 41% overall yield for the three steps (Scheme 4).

**Scheme 4:**
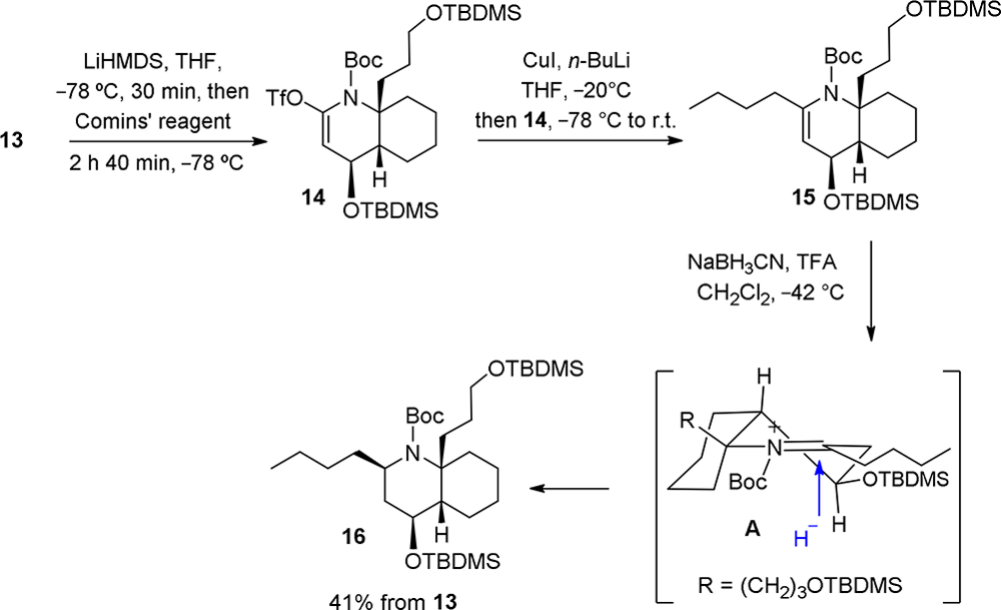
Stereoselective Introduction of the C_2_ Butyl Chain

Unfortunately, the stereochemical outcome of the reaction could not be confirmed at this stage due to the overlapping of the signals corresponding to H-2 and H-4 in the ^1^H NMR spectrum of **16**.

As mentioned before, it was envisaged that closure of the pyrrolidine C ring would be achieved by the regioselective intramolecular ring opening of an epoxide, a transformation that would also install a functionalized one-carbon appendage at C_13_. This required a previous one-carbon homologation of the silyloxypropyl chain of **16**, which was accomplished by selective deprotection of the primary alcohol with bismuth(III) triflate followed by oxidation of **17** with the Dess–Martin periodinane and subsequent Wittig methylenation. Then, deprotection of the secondary alcohol present in **18** followed by acetylation afforded intermediate **19**, with the C_4_ acetoxy substituent characteristic of cylindricine H (Scheme 5).

**Scheme 5:**
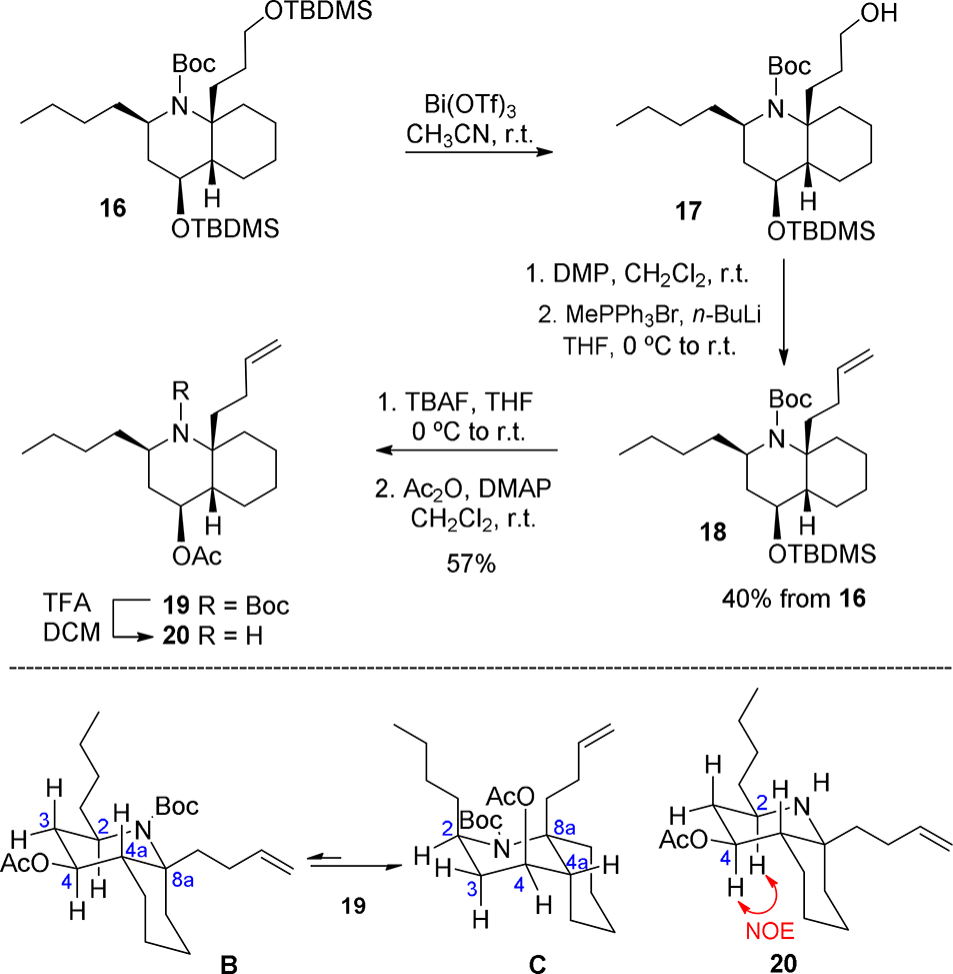
Manipulation of the C_4_ and C_8a_ DHQ Substituents and Determination of the Configuration at C_2_

The ^1^H NMR spectrum of **19** showed clear signals for H-2 and H-4 (δ 4.02 and 5.22, respectively), so at this point we decided to analyze the configurational identity of the C_2_ stereocenter. We initially assumed that the DHQ system would adopt conformation **B**, in which the three substituents on the piperidine ring would be equatorial. However, NOESY experiments did not show a NOE effect of H-2 with either H-4 or H-4a. Moreover, the multiplicity of H-4 (triplet, with two *J* of 7.5 Hz) did not agree with such a conformation **B**. These spectroscopic data led us to consider that the DHQ system would preferably adopt conformation **C** to avoid the A^1,3^ interactions of the *N*-Boc group with the equatorial chains at the C_2_ and C_8a_ positions in conformation **B**. To corroborate this hypothesis, the Boc protecting group of **19** was removed, giving rise to the secondary amine **20**, whose ^1^H NMR spectrum was in agreement with the proposed structure. A NOESY experiment showed a clear NOE effect between H-2 and H-4, thus confirming that the configuration of the C_2_ stereocenter was the one present in cylindricine H.

A Sharpless asymmetric dihydroxylation^14^ of alkene **19**, followed by ring closure, was expected to stereoselectively generate epoxide **22**, which possesses the required configuration for the synthesis of cylindricine H. Indeed, treatment of a *t*-BuOH–H_2_O solution of **19** with AD-mix-β at 0 °C afforded in excellent yield a diastereomeric mixture (78:22 ratio) of diol **21** and its C_13_ epimer (cylindricine numbering), which could not be separated by either crystallization or chromatographic methods. Mesylation of the primary alcohol followed by basic treatment gave epoxide **22** as a mixture of epimers. Subsequent removal of the Boc protecting group with TFA and quenching of the reaction mixture with aqueous KOH regioselectively provided the desired tricyclic compound **23** as a 4:1 mixture of epimers at C_13_, which could be satisfactorily separated by column chromatography. Finally, alcohol **23** was converted in excellent yield to cylindricine H by treatment with NH_4_SCN under Mitsunobu conditions,^3f^ thus accomplishing the first total synthesis of this natural product (Scheme 6). The thiocyanate analog of cylindricine J (a 6,6,6-system), arising from ring expansion of the pyrrolidine ring, was not observed. The spectroscopic data of our synthetic cylindricine H matched those previously reported for the natural product, and its optical rotation was [α]^20^_D_ = −8.5 (*c* 0.47, MeOH). Therefore, the levorotatory enantiomer has the 2*R*,4*S*,5*S*,10*S*,13*S* absolute configuration.

**Scheme 6:**
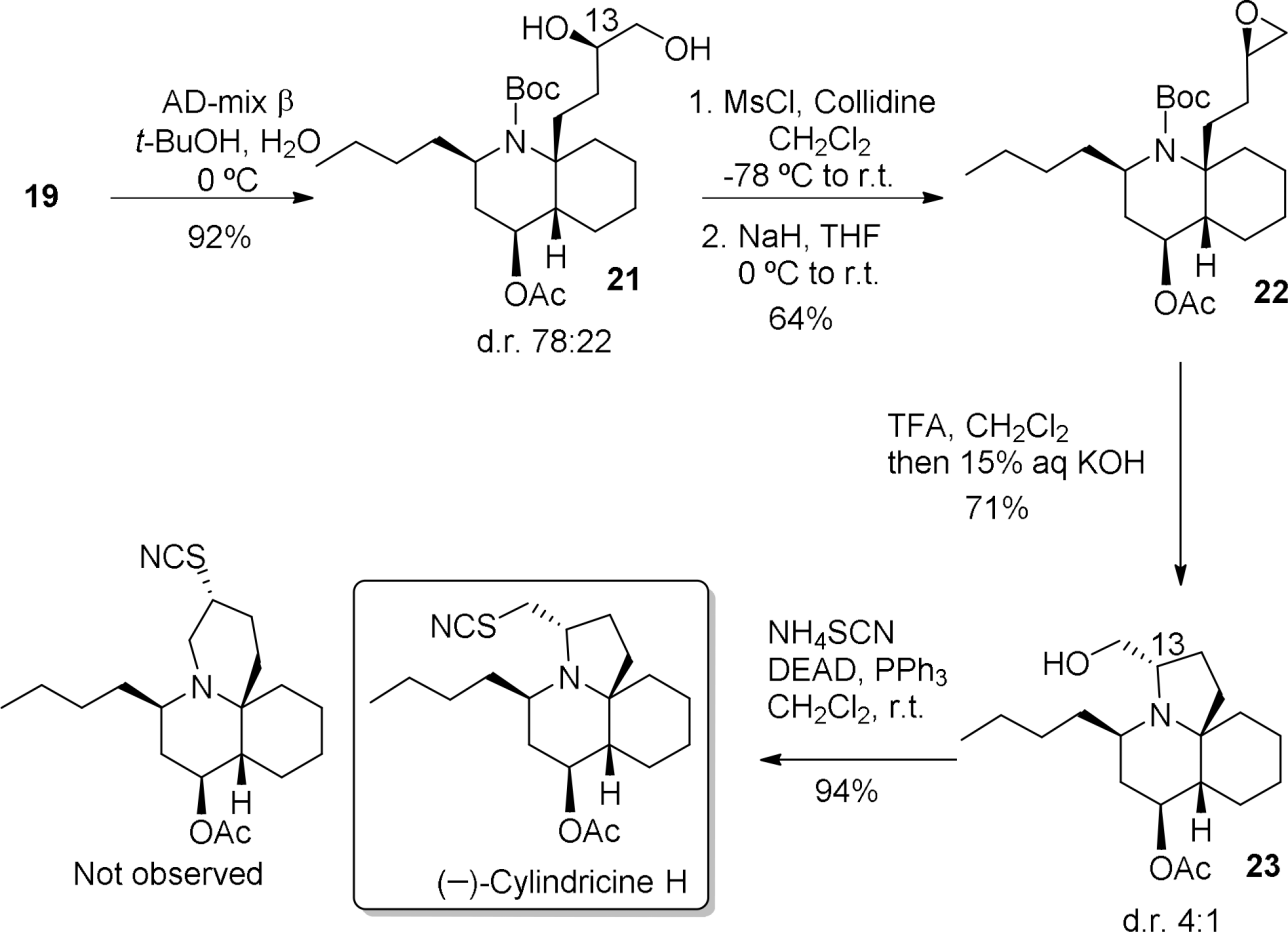
Total Synthesis of (−)-Cylindricine H

In summary, the first total synthesis of (−)-cylindricine H has been achieved by employing the tricyclic lactam **1** as the starting enantiomeric scaffold. The synthetic sequence includes as key steps the formation of the quaternary stereocenter by the insertion of an allyl substituent on an hemiaminal moiety and a series of highly stereoselective transformations for the incorporation of the C_4_ acetoxy and C_2_ butyl groups by synthetic manipulation of the amide functionality. To complete the synthesis, the pyrrolidine ring was closed by intramolecular opening of an epoxide. The synthesis of (−)-cylindricine H further illustrates the potential of phenylglycinol-derived tricyclic lactams for the assembly of complex natural products bearing the DHQ nucleus.

## Supporting Information Available

Complete experimental procedures and copies of ^1^H and ^13^C NMR spectra of all new compounds (PDF)
