# Supplementary material for: Total Synthesis of (−)-Cylindricine H
Source: Org Lett. 2022 Jul 18;24(29):5356–60. doi: 10.1021/acs.orglett.2c02004 (PMC9881165; doi:10.1021/acs.orglett.2c02004)
Supplement: Supplementary file 1 — ol2c02004_si_001.pdf [file ol2c02004_si_001.pdf]

Total Synthesis of (–)-Cylindricine H

Miriam Piccichè, Alexandre Pinto, Rosa Griera, Joan Bosch, and Mercedes Amat*

Laboratory of Organic Chemistry, Faculty of Pharmacy and Food Sciences, and Institute of Biomedicine (IBUB), University of Barcelona, 08028-Barcelona, Spain

I) Experimental procedures and spectroscopic data: pages S2-S23

II) Comparison of the 13C NMR of natural and synthetic Cylindricine H: S24-S25

III) Copies of ^{1}H and ^{13}C NMR spectra: pages S26-S47

I) Experimental procedures and spectroscopic data

General Procedures. All air sensitive reactions were performed under a dry argon or nitrogen atmosphere with dry, freshly distilled solvents using standard procedures. For reactions that require heating, a hot plate magnetic stirrer with an aluminium heating block was used. Drying of organic extracts during the work-up of reactions was performed over anhydrous Na_2SO_4 or $MgSO_4$. Evaporation of solvent was accomplished with a rotatory evaporator. Thin-layer chromatography was done on SiO_2 (silica gel 60 F_{254}), and the spots were located by UV and either a 1% $KMnO_4$ solution. Chromatography refers to flash column chromatography and was carried out on SiO_2 (silica gel 60, 230-400 mesh). NMR spectra were recorded at 400 or 500 MHz (1H) and 100.6 or 125 MHz (13C), and chemical shifts are reported in δ values, in parts per million (ppm) relative to Me_4Si (0 ppm) or relative to residual chloroform (7.26 ppm, 77.0 ppm), methanol (3.31 ppm, 49.0 ppm) or dimethylsulfoxide (2.54 ppm, 39.5 ppm) as an internal standard. Data are reported in the following manner: chemical shift, multiplicity (s = singlet, d = doublet, t = triplet, q = quartet, br = broad, m = multiplet), coupling constant (*J*) in hertz (Hz), integrated intensity, and assignment (when possible). Assignments and stereochemical determinations are given only when they are derived from definitive two-dimensional NMR experiments (HSQC-COSY). IR spectra were performed in a spectrophotometer Nicolet Avatar 320 FT-IR and only noteworthy IR absorptions (cm^{-1}) are listed. Optical rotation were measured on Perkin-Elmer 241 polarimeter. $[\alpha]_D$ values are given in 10^{-1} deg cm^2 g^{-1}. High resolution mass spectra (HMRS) were performed by *Centres Científics i Tecnològics de la Universitat de Barcelona* using an electrospray (ESI) ionization source and a TOF analyzer.

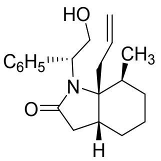

(3a*R*,7*S*,7a*R*)-1-[(1*R*)-2-Hydroxy-1-phenylethyl]-7-methyl-2-oxo-7a-(2-propenyl)octahydroindole (4): Allyltrimethylsilane (403 µL, 2.52 mmol) was added at –78 °C to a solution of **3**[1] (311 mg, 1.15 mmol) in CH_2Cl_2 (5 mL), and the mixture was stirred for 20 min. Then, $TiCl_4$ (290 µL, 2.52 mmol) was added dropwise, and the stirring was continued at –78 °C for 2 h. The mixture was poured into saturated aqueous NH_4Cl (5 mL), the organic phase was separated, and the aqueous phase was extracted with CH_2Cl_2. The combined organic extracts were dried and concentrated. Flash chromatography (from CH_2Cl_2 to 95:5 CH_2Cl_2–CH_3OH) afforded octahydroindolone **4** (270 mg, 75%) as a white solid: $[\alpha]^{22}_{D}$ +104.5 (c 1.02, $CHCl_3$); IR (film): 3366, 2930, 1662 cm^{-1}; ^{1}H-NMR (400 MHz, $CDCl_3$, COSY, *g*-HSQC) δ: 7.36-7.23 (m, 5H, H-Ar), 4.90-4.76 (m, 3H, C*H*=CH_2, CH_2=CH), 4.64-4.59 (m, 2H, C*H*N, OH), 4.29-4.22 (m, 1H, CH_2OH), 4.16-4.09 (m, 1H, CH_2OH), 2.56 (dd, *J* = 12.4, 16.0 Hz, 1H, H-3), 2.53-2.39 (m, 2H, H-3a, CH_2CH), 2.28 (dd, *J* = 7.6, 15.6 Hz, 1H, H-3), 2.25-2.19 (m, 1H, CH_2CH), 1.93-1.86 (m, 1H, H-7), 1.60-1.42 (m, 5H, H-4, H-5, H-6), 1.24-1.11 (m, 1H, H-6), 1.10 (d, *J* = 6.4 Hz, 3H, CH_3); ^{13}C-NMR (100.6 MHz, $CDCl_3$) *δ*: 177.6 (*C*O), 138.9 (C-*i*), 132.8 (*C*H=CH_2), 128.4 (2C-Ar), 127.6 (2C-Ar), 127.3 (C-Ar), 118.3 (*C*H_2=CH), 69.2 (C-7a), 66.1 (*C*H_2OH), 61.8(*C*HN), 39.0 (C-7), 37.0 (C-3a), 33.9 (C-3), 32.5 (*C*H_2CH), 32.2 (C-6), 23.8 (C-4), 19.8 (C-5), 16.6 (*C*H_3); HRMS (ESI-TOF) m/z: $[M + H]^+$ Calcd for $C_{20}H_{28}NO_2$ 314.2115; Found 314.2129.

[1] Ghirardi, E.; Griera, R.; Piccichè, M.; Molins, E.; Fernández, I.; Bosch, J.; Amat, M. Stereocontrolled Access to Enantiopure 7-Substituted *cis*- and *trans*-Octahydroindoles. *Org. Lett.* **2016**, *18*, 5836–5839.

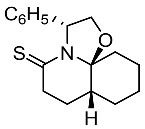

(3*R*,7a*R*,11a*S*)-3-Phenyl-5-thioperhydrooxazolo[2,3-*j*]quinoline (5): Lawesson's reagent (8.29 g, 0.02 mol, 97%) was added to a stirring solution of tricyclic lactam **1**[2] (5.4 g, 0.02 mol) in anhydrous THF (184 mL), and the mixture was heated at reflux temperature for 2 h. After cooling to room temperature, the solution was concentrated. Flash chromatography (from 98:2 to 9:1 hexane–EtOAc) afforded thiolactam **5** as a white solid (4.71 g, 82%) mp 106-108 °; $[\alpha]^{20}_{D}$ = –182 (*c* 1.0, $CHCl_3$); IR (NaCl): 1154 (C=S) cm^{-1}; ^{1}H-NMR (400 MHz, $CDCl_3$, COSY, *g*-HSQC) δ: 7.35-7.30 (m, 2H, H-Ar), 7.27-7.23 (m, 1H, H-Ar), 7.15-7.12 (m, 2H, H-Ar), 5.82 (t, *J* = 8 Hz, 1H, H-3), 4.56 (t, *J* = 8.9 Hz, 1H, H-2), 3.97 (t, *J* = 8.9 Hz, 1H, H-2), 3.22-3.17 (m, 2H, H-6), 2.12-1.99 (m, 1H), 1.98-1.88 (m, 3H), 1.74-1.70 (m, 1H), 1.69-1.59 (m, 3H), 1.55-1.45 (m, 3H); ^{13}C-NMR (100.6 MHz, $CDCl_3$) *δ*: 198.5 (NCS), 138.5 (C*q*-Ar), 128.5 (CH-Ar), 127.1 (CH-Ar), 125.4 (CH-Ar), 96.7 (C-11a), 69.1 (C-2), 64.4 (C-3), 40.3 (C-6), 38.6 (C-7a), 29.0 (C-11), 27.8 (C-7), 22.3 (C-8), 22.2 (CH_3), 19.2 (CH_2); HRMS (ESI-TOF) m/z: $[M + H]^+$ Calcd for $C_{17}H_{22}NOS$ 288.1417; Found 288.1418.

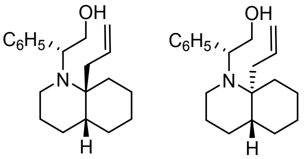

(4a*R*,8a*S*)-8a-Allyl-1-[(*R*)-2-hydroxy-1-phenylethyl]decahydroquinoline (7) and (4a*R*,8a*R*)-8a-Allyl-1-[(*R*)-2-hydroxy-1-phenylethyl]decahydroquinoline (8a-*epi*-7) : *1st step*: Methanol (12 mL, 295.7 mmol) was added dropwise via a syringe pump over a period of 6 h to a

---

[2] Amat, M.; Bassas, O.; Llor, N.; Cantó, M.; Pérez, M.; Molins, E.; Bosch, J. Dynamic Kinetic Resolution and Desymmetrization Processes: A Straightforward Methodology for the Enantioselective Synthesis of Piperidines. *Chem. Eur. J.* **2006**, *12*, 7872–7881.

refluxing solution of thiolactam **5** (1.0 g, 3.48 mmol) and $NaBH_4$ (2.1 g, 55.7 mmol) in *t*-BuOH (28 mL). Water was added to the cooled mixture and the resulting solution was extracted with CH_2Cl_2. The organic solution was dried and concentrated to give the crude amine **6**.

2nd step: AllylMgBr (7.0 mL of a 1.0 M solution in Et_2O, 7.0 mmol) was slowly added to a stirring solution of the above crude amine in anhydrous THF (18.3 mL), at –78 °C under an argon atmosphere, and the stirring was continued at this temperature for 30 min and at room temperature for 18 h. Then, saturated aqueous NH_4Cl and CH_2Cl_2 were added, the phases were separated, and the aqueous phase was extracted with CH_2Cl_2. The combined organic extracts were dried and concentrated. Flash chromatography (9.5:0.5 hexane–EtOAc), afforded compounds **7** (635 mg, 61% from **5**) and 8a-*epi*-**7** (31 mg, 3% from **5**) as yellow oils. **7** (higher R_f): $[\alpha]^{20}_D$ = –10.9 (*c* 1.0, $CHCl_3$); IR (NaCl): 3415 (OH), 3067 (C=CH), 1457 (C=C) cm^{-1}; ^{1}H-NMR (400 MHz, $CDCl_3$, COSY, *g*-HSQC) δ: 7.30-7.22 (m, 5H, H-Ar), 5.60 (ddd, *J* = 7.6, 9.8, 17.6 Hz, 1H, C*H*=CH_2), 4.90 (dd, *J* = 1.2, 10.0 Hz, 1H, CH=C*H*$_2$), 4.82 (d, *J* = 18.0 Hz, 1H, CH=C*H*$_2$), 4.27 (dd, *J* = 6.0, 10.8 Hz, 1H, H-1’), 3.87 (t, *J* = 10.6 Hz, 1H, H-2’), 3.70 (br s, 1H, OH), 3.48-3.43 (m, 1H, H-2’), 3.06-3.02 (m, 1H, H-2), 2.81 (td, *J* = 3.2, 12.0 Hz, 1H, H-2), 2.52 (dd, *J* = 7.6, 13.8 Hz 1H, H-1’’), 2.14-2.08 (m, 1H), 2.05-1.98 (m, 1H), 1.91-1.73 (m, 3H), 1.72-1.63 (m, 1H), 1.53-1.45 (m, 4H, H-4a), 1.32 (dd, *J* = 7.2, 13.6 Hz, 1H, H-1’’), 1.27-1.20 (m, 3H); ^{13}C-NMR (100.6 MHz, $CDCl_3$) δ: 139.5 (C*q*-Ar), 135.2 (*C*=CH_2), 129.1 (CH-Ar), 128.2 (CH-Ar), 127.5 (CH-Ar), 116.9 (C=*C*H_2), 61.2 (C-2’), 59.4 (C-8a), 59.0 (C-1’), 39.2 (C-2), 37.6 (C-4a), 36.7 (C-1’’), 33.1 (CH_2), 27.1 (CH_2), 26.4 (CH_2), 25.5 (CH_2), 21.4 (CH_2), 20.7 (CH_2); HRMS (ESI-TOF) m/z: $[M + H]^+$ Calcd for $C_{20}H_{30}NO$ 300.2332; Found 300.2330. 8a-*epi*-**7** (lower R_f): $[\alpha]^{20}_D$ = –83.1 (*c* 1.04, $CHCl_3$); IR (NaCl): 3417, 2931 cm^{-1}; ^{1}H-NMR (400 MHz, $CDCl_3$) δ: 7.38-7.30 (m,

5H, H-Ar), 5.98-5.86 (m, 1H, C*H*=CH_2), 5.19 (d, *J* = 13.6 Hz, 2H, CH=CH_2), 4.38-4.36 (m, 1H), 3.93 (t, *J* = 8.4 Hz, 1H), 3.48-3.38 (m, 1H), 3.18 (br s, 1H, OH), 3.06-2.94 (m, 2H), 2.78-2.72 (m, 1H), 2.35 (dd, *J* = 4.8, 13.8 Hz, 1H), 1.92-1.86 (m, 1H), 1.78-1.70 (m, 3H), 1.64-1.57 (m, 1H), 1.38-1.21 (m, 5H), 1.19-1.06 (m, 2H), 0.75-0.68 (m, 1H); ^{13}C-NMR (100.6 MHz, $CDCl_3$) δ: 142.2 (C*q*-Ar), 135.1 (*C*=CH_2), 128.4 (CH-Ar), 128.3 (CH-Ar), 127.1 (CH-Ar), 117.7 (C=*C*H_2), 61.5 (C-2'), 59.7 (C-8a), 58.4 (C-1'), 43.8 (C-4a), 38.5 (C-2), 33.7 (CH_2), 30.5 (CH_2), 30.0 (CH_2), 27.0 (CH_2), 26.1 (CH_2), 24.3 (CH_2), 23.0 (CH_2); HRMS (ESI-TOF) m/z: $[M + H]^+$ Calcd for $C_{20}H_{30}NO$ 300.2332; Found 300.2331.

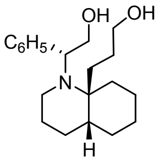

(4a*R*,8a*S*)-1-[(*R*)-2-Hydroxy-1-phenylethyl]-8a-(3-hydroxypropyl) decahydroquinoline (9): BH_3·THF (7.25 mL of a 1.0 M solution in THF, 7.25 mmol) was added dropwise to a stirring solution of the compound **7** (1.67 g, 5.57 mmol) in anhydrous THF (55.7 mL) at 0 °C, and the mixture was stirred at 60 °C for 2 h. Then, 3 M aqueous NaOH (18.5 mL) and 30% aqueous H_2O_2 (18.5 mL) were added sequentially at 0 °C, and the mixture was stirred at 70 °C for 2 h. After cooling to room temperature, saturated aqueous K_2CO_3 was added, the phases were separated, and aqueous phase was extracted with EtOAc. The combined organic extracts were dried and concentrated. Flash chromatography (KP-NH Biotage® SNAP cartridge, 3:7 hexane–EtOAc) afforded compound **9** (1.72 g, 97%) as a white solid: mp 117-119 °C; $[\alpha]^{20}_D$ = +10.15 (*c* 1.05, MeOH);IR (NaCl): 3392, 3400 cm^{-1}; ^{1}H-NMR (400MHz, CD_3OD, COSY, *g*-HSQC) δ: 7.40 (d, *J* = 6.8 Hz, 2H, H-Ar), 7.34-7.30 (m, 2H, H-Ar), 7.28-7.24 (m, 1H, H-Ar), 4.27 (t, *J* = 7.4 Hz, 1H, H-3), 3.97 (dd, *J* = 2.0, 8.2 Hz, 1H, H-2), 3.65-3.58 (m, 1H, H-2), 3.36-

3.33 (m, 1H), 3.29-3.20 (m, 2H), 3.06-3.02 (m, 1H), 2.94-2.82 (m, 1H), 2.19-2.14 (m, 1H), 1.98 (br s, 1H, OH), 1.86-1.67 (m, 4H), 1.64-1.58 (m, 1H), 1.53-1.46 (m, 3H), 1.40-1.25 (m, 5H); ^{13}C-NMR (100.6 MHz, CD_3OD) δ: 142.1 (C*q*-Ar), 130.1 (CH-Ar), 129.1 (CH-Ar), 128.0 (CH-Ar), 64.3 (CH), 63.6 (2 carbons CH_2), 60.7 (C-3), 60.2 (C-8a), 41.5 (CH_2), 38.5 (C-4a), 33.1 (CH_2), 29.2 (CH_2), 28.7 (CH_2), 28.2 (CH_2), 27.1 (CH_2), 23.5 (CH_2), 22.6 (CH_2); HRMS (ESI-TOF) m/z: $[M + H]^+$ Calcd for $C_{20}H_{32}NO_2$ 318.2428; Found 318.2423.

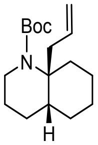

(4a*R*,8a*S*)-8a-Allyl-1-(*tert*-butoxycarbonyl)decahydroquinoline: *1st step*: A solution of **9** and Boc_2O (200 mg, 0.63 mmol) in EtOAc (1.5 mL) containing $Pd(OH)_2$ (80 mg) was stirred under hydrogen at room temperature for 24 h. The catalyst was removed by filtration over Celite® and the filtrate was concentrated under reduced pressure affording the crude carbamate.

2nd step: n-Bu_3P (187 µL, 0.75 mmol) was added dropwise to a stirring solution of the above crude and *o*-nitrophenyl selenocyanate (208 mg, 0.75 mmol) in anhydrous THF (2.1 mL) at room temperature. After 2 h, H_2O_2 (790 µL) was added, and the resulting mixture was stirred for 14 h at room temperature. H_2O was added, the layers were separated, and the aqueous phase was extracted with CH_2Cl_2. The combined organic extracts were dried and concentrated. Flash chromatography (7:3 hexane–EtOAc) afforded the title compound (63 mg, 36% from **9**) as a colorless oil: $[\alpha]^{20}_D$ = +45.05 (*c* 1.0, $CHCl_3$); IR (NaCl): 2929, 1695 cm^{-1}; ^{1}H-NMR (400 MHz, $CDCl_3$, COSY, *g*-HSQC) δ: 5.86-5.76 (m, 1H, C*H*=CH_2), 5.10-5.03 (m, 2H, CH=CH_2), 3.87 (t, *J* = 13.8, 4.7 Hz, 1H), 3.16-3.08 (m, 1H), 2.73-2.62 (m, 2H, H-1'), 2.30 (br s, 1H, H-2), 1.84-1.63 (m, 4H), 1.60-1.45 (m, 5H), 1.44 [s, 9H, $OC(CH_3)_3$], 1.37-1.20 (m, 3H); ^{13}C-NMR (100.6 MHz, $CDCl_3$) δ: 156.2 (CO),

134.5 (C=CH_2), 117.4 (C=CH_2), 79.1 [OC(CH_3)$_3$], 60.6 (C-8a), 41.5 (CH_2), 39.3 (C-1'), 36.7 (C-4a), 33.5 (C-2), 28.5 [OC(CH_3)$_3$], 28.2 (CH_2), 24.2 (CH_2), 22.9 (CH_2), 22.7 (CH_2), 21.5 (CH_2); HRMS (ESI-TOF) m/z: $[M + H]^+$ Calcd for $C_{17}H_{30}NO_2$ 280.2271; Found 280.2278.

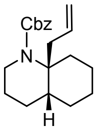

(4a*R*,8a*S*)-8a-Allyl-1-(benzyloxycarbonyl)decahydroquinoline (8): *First step*: TFA (170 µL, 2.2 mmol) was added to a stirred solution of (4a*R*,8a*S*)-8a-alyl-1-(*tert*-butoxycarbonyl)decahydroquinoline (63 mg, 0.22 mmol) in CH_2Cl_2 (2.2 mL). After 1 h, the solvent was evaporated, and the residue was taken up with CH_2Cl_2. The organic solution was sequentially washed with saturated aqueous $NaHCO_3$ and brine, dried and concentrated. The resulting crude secondary amine was used in the next step without further purification.

2nd step: CbzCl (30 µL, 0.26 mmol) was added dropwise to a stirring solution of the above crude residue and K_2CO_3 (60 mg, 0.44 mmol) in anhydrous CH_2Cl_2 (500 µL) at room temperature. After 14h, H_2O was added, the layers were separated, and the aqueous phase was extracted with CH_2Cl_2. The combined organic extracts were dried and concentrated. Flash chromatography (9:1 hexane–EtOAc) afforded compound **8** (42 mg, 61% for the two steps) as a colorless oil: ^{1}H-NMR (400 MHz, DMSO-d_6) δ: 7.39-7.28 (m, 5H), 5.78-5.67 (m, 1H), 5.05-4.96 (m, 4H), 3.77 (dt, J = 13.2, 4.7 Hz, 1H), 3.21-3.14 (m, 1H), 2.71-2.60 (m, 1H), 2.32 (br s, 1H), 1.80-1.61 (m, 4H), 1.58-1.44 (m, 5H), 1.43-1.26 (m, 3H).

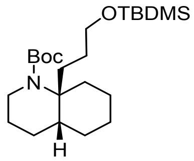

(4a*R*,8a*S*)-1-(*tert*-Butoxycarbonyl)-8a-[3-(*tert*-butyldimethylsilyloxy)propyl]decahydroquinoline (10): *1st step*: A suspension of **9** (1.17 g, 3.68 mmol) in 1.25 M HCl in MeOH (61 mL) containing $Pd(OH)_2$ (468 mg) was stirred under hydrogen at room temperature for 24 h. The catalyst was removed by filtration and the filtrate was concentrated. The residue was taken up in CH_2Cl_2 and 2 M aqueous HCl, the layers were separated, and the aqueous phase was washed with CH_2Cl_2. The aqueous phase was then basified with solid KOH and extracted with EtOAc. The combined organic extracts were dried and concentrated.

2nd step: TEA (667 µL, 4.78 mmol), DMAP (23 mg, 0.184 mmol) and TBDMSCl (665 mg, 4.42 mmol) were added at room temperature to a stirring solution of the above crude secondary amine in anhydrous THF (18.4 mL). After 20 h, 5% aqueous KOH was added and the layers were separated. The aqueous phase was extracted with CH_2Cl_2. The combined organic extracts were dried and concentrated.

3rd step: Di-*tert*-butyl dicarbonate (1.2 g, 5.52 mmol) was added to a stirring solution of the above crude silyl derivative and DMAP (45 mg, 0.368 mmol) in anhydrous CH_2Cl_2 (13 mL) at 0 °C. After stirring overnight at room temperature, the mixture was diluted with CH_2Cl_2 and saturated aqueous NH_4Cl was added. The phases were separated, and the aqueous phase was extracted with CH_2Cl_2. The combined organic extracts were dried and concentrated. Flash chromatography (from 99:1 to 95:5 hexane–EtOAc) afforded **10** (940 mg, 62% from **9**) as a yellow oil: $[\alpha]^{20}_{D}$ = + 18.24 (*c* 1.525, $CHCl_3$); IR (NaCl): 1699 cm^{-1}; ^{1}H-

NMR (400 MHz, $CDCl_3$, COSY, *g*-HSQC) δ: 3.88-3.81 (m, 1H, H-2), 3.64-3.52 (m, 2H, CH_2OSi), 3.09-3.02 (m, 1H, H-2), 2.48-2.40 (m, 1H), 2.07 (ddd, *J* = 4.6, 8.9, 21.6 Hz, 1H), 1.77-1.56 (m, 8H), 1.51-1.39 (m, 15H), 1.35-1.26 (m, 1H), 0.89 [s, 9H, $SiC(CH_3)_3$], 0.05 [s, 6H, $Si(CH_3)_2$]; ^{13}C-NMR (100.6 MHz, $CDCl_3$) *δ*: 156.4 (CO), 79.0 [O*C*$(CH_3)_3$], 63.7 (*C*H_2OSi), 60.5 (C-8a), 41.6 (C-2), 37.8 (C-4a), 34.4 (CH_2), 31.8 (CH_2), 28.5 [OC(*C*$H_3)_3$], 28.4 (CH_2), 26.8 (CH_2), 26.0 [SiC(*C*$H_3)_3$], 24.7 (CH_2), 23.6 (CH_2), 22.9 (CH_2), 21.4 (CH_2), 18.3 [Si*C*$(CH_3)_3$], –5.2 [$Si(CH_3)_2$]; HRMS (ESI-TOF) m/z: $[M + H]^+$ Calcd for $C_{23}H_{46}NO_3Si$ 412.3241; Found 412.3248.

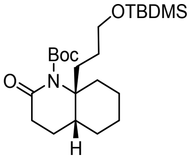

(4a*R*,8a*S*)-1-(*tert*-Butoxycarbonyl)-8a-[3-(*tert*-butyldimethylsilyloxy)propyl]-2-oxodecahydroquinoline (11): $RuO_2 \cdot nH_2O$ (8.8 mg, 0.066 mmol) was added at room temperature to a stirring heterogeneous mixture of **10** (900 mg, 2.19 mmol) in EtOAc (4.4 mL) and 10% aqueous $NaIO_4$ (11 mL). After stirring for 2 h, the phases were separated, and the aqueous phase was extracted with EtOAc. 2-Propanol was added to the combined organic extracts, the resulting suspension was filtered over Celite®, and the filtrate was evaporated. Flash chromatography (from 95:5 to 8:2 hexane–EtOAc) afforded lactam **11** as a yellowish oil (764 mg, 82 %): $[\alpha]^{20}_D$ = +8.98 (c 1.385, $CHCl_3$); IR (NaCl): 1699, 1740 cm^{-1}; ^{1}H-NMR (400 MHz, $CDCl_3$, COSY, *g*-HSQC) δ: 3.58 (t, *J* = 6.4 Hz, 2H, CH_2OSi), 2.54-2.45 (m, 1H, H-3), 2.41-2.33 (m, 1H, H-3), 2.15-2.07 (m, 1H), 2.02-1.91 (m, 1H), 1.88-1.79 (m, 2H, H-4a), 1.70-1.58 (m, 7H), 1.51 [s, 9H, $OC(CH_3)_3$], 1.54-1.45 [m, 3H), 1.38-1.26 (m, 1H), 0.89 [s, 9H, $SiC(CH_3)_3$], 0.04 [s, 6H, $Si(CH_3)_2$]; ^{13}C-NMR (100.6 MHz2, $CDCl_3$) δ: 171.5 (NCO), 154.0 (COO), 83.3 [O*C*$(CH_3)_3$], 63.3 (C-8a), 63.2 (CH_2OSi), 35.5 (CH_2), 35.1 (C-4a),

33.2 (CH_2), 29.0 (C-3), 27.6 [COOC(CH_3)$_3$], 27.4 (CH), 26.8 (CH_2), 25.9 [SiC(CH_3)$_3$], 24.2 (CH_2), 22.1 (CH_2), 21.9 (CH_2), 18.3 (Si-C*q*), –5.3 [Si(CH_3)$_2$]; HRMS (ESI-TOF) m/z: $[M + H]^+$ Calcd for $C_{23}H_{44}NO_4SiNa$ 448.2854; Found 448.2857.

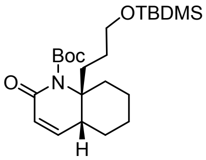

(4a*R*,8a*S*)-1-(*tert*-Butoxycarbonyl)-8a-[3-(*tert*-butyldimethyl-silyloxy)propyl]-2-oxo-1,2,4a,5,6,7,8,8a-octahydroquinoline (12): *1st step*: LiHMDS (5.0 mL of a 1 M solution in THF, 5.0 mmol) was added to a solution of compound **11** (1.02 g, 2.39 mmol) in anhydrous THF (11 mL) at –78 °C, and the mixture was stirred at this temperature for 1 h and 20 min. Then, a solution of PhSeCl (503 mg, 2.63 mmol) in anhydrous THF (13.2 mL) was added dropwise, and the stirring was continued at –78 °C for 1 h and at room temperature for 30 min. Saturated aqueous $NaHCO_3$ was added and the aqueous phase was extracted with EtOAc. The organic extracts were washed with saturated aqueous $NaHCO_3$, dried and concentrated.

2nd step: H_2O_2 (470 μL, 15.3 mmol) was added to a stirring solution of the above crude seleno derivatives and pyridine (212 μL, 2.6 mmol) in anhydrous CH_2Cl_2 (120 mL) at 0 °C. Then, the stirring was continued at room temperature for 1.5 h. H_2O was added, and the mixture was diluted with CH_2Cl_2. The phases were separated, and the aqueous phase was extracted with CH_2Cl_2. The combined organic extracts were dried and concentrated. Flash chromatography (from hexane to 9:1 hexane–EtOAc) afforded **12** (881 mg, 87%) as a yellow oil: $[\alpha]^{20}_D$ = –60.63 (c 1.0, $CHCl_3$); IR (NaCl): 1737, 1682, 1623 cm^{-1}; ^1H-NMR (400 MHz, $CDCl_3$, COSY, *g*-HSQC) δ: 6.59 (dd, *J* = 5.2, 9.6 Hz, 1H, H-4); 5.87 (d, *J* = 9.6 Hz, 1H, H-3), 3.54 (t, *J* = 6.4 Hz, 2H, CH_2OSi), 2.66-2.29 (m, 1H), 2.46-2.34 (m, 1H), 2.21-

2.18 (m, 1H), 2.03-1.95 (m, 1H), 1.83-1.74 (m, 2H), 1.69-1.65 (m, 1H), 1.51 (s, 9H), 1.59-1.42 (m, 4H), 1.40-1.24 (m, 2H), 0.88 (s, 9H, $3CH_3$), 0.03 (s, 6H, $2CH_3$); ^{13}C-NMR (100.6 MHz, $CDCl_3$) δ: 164.6 (C-2); 153.6 (NCO), 146.2 (C-4), 123.6 (C-3), 83.1 [O*C*(CH_3)$_3$], 63.2 (C-8a), 62.8 (CH_2OSi), 39.3 (C-4a), 31.8 (CH_2), 28.7 (CH_2), 28.3 (CH_2), 27.7 [OC(*C*H_3)$_3$], 25.9 [SiC(*C*H_3)$_3$], 24.4 (CH_2), 21.5 (CH_2), 18.2 (Si-C*q*), –5.3 [Si(CH_3)$_2$]; HRMS (ESI-TOF) m/z: $[M + Na]^+$ Calcd for $C_{23}H_{41}NO_4SiNa$ 446.2697; Found 446.2700.

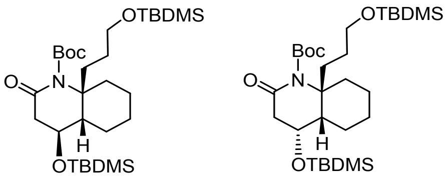

(4*S*,4a*S*,8a*S*)-1-(*tert*-Butoxycarbonyl)-4-(*tert*-butyldimethylsilyloxy)-8a-[3-(*tert*-butyldimethylsilyloxy)propyl]-2-oxodecahydroquinoline (13) and (4*R*,4a*S*,8a*S*)-1-(*tert*-Butoxycarbonyl)-4-(*tert*-butyldimethylsilyloxy)-8a-[3-(*tert*-butyldimethylsilyloxy)propyl]-2-oxodecahydroquinoline (4-*epi*-13): *1st step*: A suspension of CuCl (14 mg, 0.145 mmol), dppbz (65 mg, 0.145 mmol) and NaO*t*-Bu (29 mg, 0.29 mmol) in anhydrous THF (4.8 mL) was stirred at room temperature for 30 min. Then, a solution of B_2Pin_2 (673 mg, 2.7 mmol) in anhydrous THF (2.5 mL) was added and the resulting yellow suspension was stirred for 10 min. A solution of compound **12** (1.2 g, 2.41 mmol) in anhydrous THF (6.9 mL) and anhydrous MeOH (195 µL, 4.8 mmol) was added and the resulting brown solution was stirred at room temperature for 3 h. $NaBO_3 \cdot H_2O$ (509 mg, 5.1 mmol) and H_2O (14.2 mL) were added and stirring was continued for 2 h. Then, H_2O was added, and the aqueous phase was extracted with EtOAc. The organic extracts were washed with brine, dried and concentrated.

2nd step: TBDMSCl (741 mg, 4.8 mmol) was added to a solution of the above crude alcohol and imidazole (984 mg, 14.5 mmol) in anhydrous DMF (4.8 mL), and the mixture was stirred at room temperature overnight. Saturated aqueous $NaHCO_3$ was added, and the resulting mixture was extracted with EtOAc. The combined organic extracts were dried and concentrated. Flash chromatography (9:1 hexane–EtOAc) afforded compounds **13** (911 mg, 68%) and 4-*epi*-**13** (107 mg, 8%) as colorless oils. 4-*epi*-**13** (higher R_f): $[\alpha]^{20}_D$ = +12.2 (*c* 0.24, $CHCl_3$); IR (NaCl): 1689, 1740 cm^{-1}; ^1H-NMR (400 MHz, $CDCl_3$, COSY, *g*-HSQC) δ: 4.27 (ddd, *J* = 4.4, 7.2, 10.2 Hz, 1H, H-4), 3.64-3.51 (m, 2H, H-3'), 2.60 (dd, *J* = 7.2, 18.0 Hz, 1H, H-3), 2.44 (dd, *J* = 10.2, 18.0 Hz, 1H, H-3), 2.08-2.00 (m, 1H), 1.89-1.77 (m, 4H, H-4a), 1.73-1.65 (m, 1H), 1.58-1.45 (m, 14H), 1.31-1.15 (m, 2H), 0.88 [s, 9H, $SiC(CH_3)_3$], 0.87 [s, 9H, $SiC(CH_3)_3$], 0.06 (s, 3H, $SiCH_3$), 0.05 (s, 3H, $SiCH_3$), 0.04 [s, 6H, $Si(CH_3)_2$]; ^{13}C-NMR (100.6 MHz, $CDCl_3$) δ: 170.9 (COO), 153.8 (NCO), 83.5 [O*C*($CH_3)_3$], 63.7 (C-4), 63.0 (CH_2OSi), 62.1 (C-8a), 43.0 (C-4a), 38.1 (CH_2), 37.7 (CH_2), 33.1 (CH_2), 28.0 (CH_2), 27.6 [OC($CH_3)_3$], 25.9 [SiC(*C*$H_3)_3$], 25.7 [SiC(*C*$H_3)_3$], 25.1 (CH_2), 21.6 (CH_2), 19.5 (CH_2), 18.3 (Si-C*q*), 17.9 (Si-C*q*), –4.6 [Si(*C*$H_3)_2$], –4.7 [Si(*C*$H_3)_2$], –5.3 [Si(*C*$H_3)_2$], –5.4 [Si(*C*$H_3)_2$]; HRMS (ESI-TOF) m/z: $[M + Na]^+$ Calcd for $C_{29}H_{57}NO_5Si_2Na$ 578.3667; Found 578.3670. **13** (lower R_f): $[\alpha]^{20}_D$ = +13.1 (*c* 0.44 in $CHCl_3$); IR (NaCl): 1668, 1749 cm^{-1}; ^1H-NMR (400 MHz, $CDCl_3$, COSY, *g*-HSQC) δ: 4.12 (ddd, *J* = 5.6, 9.6, 9.6 Hz, 1H, H-4), 3.70-3.65 (m, 1H, H-3'), 3.55-3.49 (m, 1H, H-3'), 2.74 (dd, *J* = 5.6, 16.8 Hz, 1H, H-3), 2.35 (dd, *J* = 9.6, 16.8 Hz, 1H, H-3), 2.28-2.20 (m, 1H), 2.08-1.99 (m, 2H), 1.86-1.82 (m, 1H, H-4a), 1.71-1.61 (m, 3H), 1.52-1.47 (m, 10H), 1.46-1.37 (m, 5H), 0.89 [s, 9H, $SiC(CH_3)_3$], 0.88 [s, 9H, $SiC(CH_3)_3$], 0.09 (s, 3H, $SiCH_3$), 0.08 (s, 3H, $SiCH_3$), 0.05 [s, 6H, $Si(CH_3)_2$]; ^{13}C-NMR (100.6 MHz, $CDCl_3$) δ: 169.7 (COO), 153.6 (NCO), 83.6 [O*C*($CH_3)_3$], 64.3 (C-4), 63.2 (C-8a), 63.0 (CH_2OSi), 42.2 (C-3), 42.0 (C-4a), 35.7 (CH_2), 33.1 (CH_2),

27.6 [OC(CH_3)$_3$], 26.5 (CH_2), 25.9 [SiC(CH_3)$_3$], 25.7 [SiC(CH_3)$_3$], 22.7 (CH_2), 22.0 (CH_2), 20.3 (CH_2), 18.3 (Si-C*q*), 18.0 (Si-C*q*), –4.2 [Si(CH_3)$_2$], –4.9 [Si(CH_3)$_2$], –5.3 [Si(CH_3)$_2$], –5.3 [Si(CH_3)$_2$]; HRMS (ESI-TOF) m/z: $[M + Na]^+$ Calcd for $C_{29}H_{57}NO_5Si_2Na$ 578.3667; Found 578.3666.

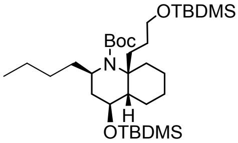

(2*R*,4*S*,4a*S*,8a*S*)-1-(*tert*-Butoxycarbonyl)-2-butyl-4-(*tert*-butyldimethylsilyloxy)-8a-[3-(*tert*-butyldimethylsilyloxy)propyl]decahydro-quinoline (16): *1st step*: LiHMDS (1.2 mL of a 1 M solution in THF, 1.22 mmol) was added to a solution of lactam **13** (400 mg, 0.72 mmol) in anhydrous THF (7.2 mL) at –78 °C, and the mixture was stirred at this temperature for 30 min. Then, a solution of Comins' reagent (594 mg, 1.44 mmol, 95%) in anhydrous THF (7.2 mL) was added, and the mixture was stirred at –78 °C for 2 h 40 min. After this time, the resulting mixture was diluted with Et_2O and concentrated under reduce pressure without heating to give crude triflate **14.**

2nd step: *n*-BuLi (3.8 mL of a 1.89 M solution in hexane, 7.2 mmol) was added to a suspension of CuI (713 mg, 3.67 mmol, 98%) in anhydrous THF (18.3 mL) at –20 °C, and the mixture was stirred at this temperature for 30 min. After cooling at –78 °C, a solution of the above triflate in anhydrous THF (36 mL) was added, and the resulting mixture was stirred at room temperature for 17 h. Hexane was added, and the resulting suspension was filtered over Celite® and the filtrate was concentrated to give crude enecarbamate **15**.

3rd step: $NaBH_3CN$ (285 mg, 4.31 mmol, 95%) was added to a stirring solution of the above carbamate in anhydrous CH_2Cl_2 (360 mL), and the stirring was continued for 15 min. Then, the mixture was cooled to –42 °C, TFA (360 µL) was

slowly added, and the stirring was continued for 2 h. A solution of saturated aqueous $NaHCO_3/H_20$ (1:1) (150 mL) was added, and the biphasic mixture was stirred for 10 min. The mixture was extracted with EtOAc, and the combined organic extracts were washed with brine, dried and concentrated. Flash chromatography (95:5 hexane–EtOAc) afforded compound **16** (176 mg, 41% yield from **13**) as a yellow oil: $[\alpha]^{20}_D$ = +27.2 (*c* 0.64, $CHCl_3$); IR (NaCl): 1691 cm^{-1}; ^{1}H-NMR (400 MHz, $CDCl_3$, COSY, *g*-HSQC) δ: 4.02 (t, *J* = 7.8 Hz, 1H, H-4), 4.00-3.94 (m, 1H, H-2), 3.72-3.62 (m, 1H, CH_2OSi), 3.59-3.53 (m, 1H, CH_2OSi), 2.44 (d, *J* = 12.8 Hz, 1H), 2.22 (td, *J* = 4.5, 13.2 Hz, 1H), 2.07-2.00 (m, 2H), 1.99-1.91 (m, 1H), 1.81 (dd, *J* = 2.0, 14.2 Hz, 1H), 1.73-1.54 (m, 4H), 1.50-1.40 [m, 13H, included 1s for $O(CH_3)_3$], 1.38-1.28 (m, 5H), 1.26-1.20 (m, 2H), 0.90-0.88 [m, 21H, $2SiC(CH_3)_3$, CH_3], 0.07 (s, 3H, $SiCH_3$), 0.05 [s, 6H, $Si(CH_3)_2$], 0.04 (s, 3H, $SiCH_3$); ^{13}C-NMR (100.6 MHz, $CDCl_3$) δ: 155.0 (NCO), 78.8 [O*C*$(CH_3)_3$], 65.5 (C-4), 63.8 (CH_2OSi), 59.7 (C-8a), 51.9 (C-2), 41.2 (C-4a), 36.6 (CH_2), 33.6 (CH_2), 33.5 (CH_2), 29.9 (CH_2), 28.7 (CH_2), 28.6 [$OC(CH_3)_3$], 27.9 (CH_2), 26.0 [$SiC(CH_3)_3$], 25.7 [$SiC(CH_3)_3$], 23.9 (CH_2), 22.8 (CH_2), 21.9 (CH_2), 19.8 (CH_2), 18.4 (Si-C*q*), 17.8 (Si-C*q*), 14.2 (CH_3), –4.0 [$Si(CH_3)_2$], –5.1 [$Si(CH_3)_2$], –5.2 [$Si(CH_3)_2$], –5.2 [$Si(CH_3)_2$]; HRMS (ESI-TOF) m/z: $[M + H]^+$ Calcd for $C_{33}H_{68}NO_4Si_2$ 598.4681; Found 598.4678.

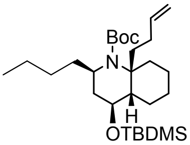

(2*R*,4*S*,4a*S*,8a*S*)-8a-(3-Butenyl)-1-(*tert*-butoxycarbonyl)-2-butyl-4-(*tert*-butyldimethylsilyloxy)decahydroquinoline (18) : *1st step*: H_2O (40 µL, 2.2 mmol) and $Bi(OTf)_3$ (22 mg, 0.035 mmol) were successively added to a stirring solution of **16** (260 mg, 0.44 mmol) in MeCN (4 mL) at room temperature. After 4.5 h, the mixture was concentrated under reduce pressure.

2nd step: Dess-Martin periodinane (246 mg, 0.58 mmol) was added at room temperature to a stirring solution of the above crude alcohol (201 mg, 0.42 mmol) in anhydrous CH_2Cl_2 (4.2 mL). After 1.5 h, 10% aqueous NaOH was added and the resulting mixture was extracted with CH_2Cl_2. The organic extracts were dried and concentrated to give a crude aldehyde.

3rd step: *n*-BuLi (693 µL of a 1.37 M solution in hexane, 0.95 mmol) was added at 0 °C to a stirring solution of methyltriphenylphosphonium bromide (378 mg, 1.04 mmol, 98%) in anhydrous THF (3.5 mL), and the resulting mixture was stirred for 1.5 h. Then, a solution of the above aldehyde in anhydrous THF (1.4 mL) was added and the mixture was stirred at room temperature for 16 h. A saturated solution of NH_4Cl was added at 0 °C and the resulting mixture was extracted with CH_2Cl_2. The organic extracts were dried and concentrated. Flash chromatography (from 95:5 to 8:2 hexane–CH_2Cl_2) afforded compound **18** (80 mg, 40% yield from **16**) as a colorless oil: $[\alpha]^{20}_D$ = + 35.01 (*c* 0.85, $CHCl_3$); IR (NaCl): 1693 cm^{-1}; ^{1}H-NMR (400 MHz, $CDCl_3$, COSY, *g*-HSQC) δ: 5.89-5.80 (m, 1H, *H*C=CH_2), 5.03 (dd, *J* = 2.0, 17.2 Hz, 1H, HC=C*H_2*), 4.93 (dd, *J* = 2.0, 10.4 Hz, 1H, HC=C*H_2*), 4.05-3.95 (m, 2H, H-2, H-4), 2.44-2.32 (m, 2H), 2.07-2.00 (m, 2H, H-4a), 1.96-1.86 (m, 2H), 1.82 (dd, *J* = 2.6, 15.0 Hz, 1H), 1.71-1.62 (m, 3H), 1.60-1.56 (m, 1H), 1.44 [br s, 12H, $OC(CH_3)_3$], 1.36-1.17 (m, 6H), 0.92-0.86 [m, 12H, $SiC(CH_3)_3$, CH_3], 0.07 (s, 3H, $SiCH_3$), 0.04 (s, 3H, $SiCH_3$); ^{13}C-NMR (100.6 MHz, $CDCl_3$) δ: 154.0 (NCO), 138.4 (H*C*=CH_2), 113.1 (HC=*C*H_2), 78.0 (C*q*-Boc), 64.5 (C-4), 58.7 (C-8a), 50.9 (C-2), 40.3 (C-4a), 35.5 (CH_2), 32.5 (CH_2), 32.5 (CH_2), 31.2 (CH_2), 28.9 (CH_2), 27.7 (CH_2), 27.6 [$O(CH_3)_3$], 24.8 [$Si(CH_3)_3$], 22.9 (CH_2), 21.8 (CH_2), 20.8 (CH_2), 18.7 (CH_2), 16.8 (Si-C*q*), 13.2 (CH_3), −5.0 ($SiCH_3$), −6.0 ($SiCH_3$); HRMS (ESI-TOF) m/z: $[M + H]^+$ Calcd for $C_{28}H_{54}NO_3Si$ 480.3867; Found 480.3873.

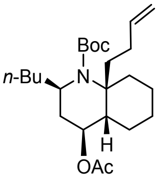

(2*R*,4*S*,4a*S*,8a*S*)-4-(Acetoxy)-8a-(3-butenyl)-1-(*tert*-butoxycarbonyl)-2-butyldecahydroquinoline (19): *1st step*: TBAF (1.0 mL of a 1 M solution in THF, 1.052 mmol) was added at 0 °C to a stirring solution of **18** (262 mg, 0.53 mmol) in anhydrous THF (5.3 mL), and the resulting mixture was stirred for 15 h at room temperature. Saturated aqueous solution of NH_4Cl was added, the resulting mixture was extracted with EtOAc, and the organic extracts were dried and concentrated. To remove the tetrabutylammonium salts the residue was taken in Et_2O/H_2O 1:1 (5 mL), the organic phase was washed with saturated aqueous NH_4Cl and the organic solution was dried and concentrated.

2nd step: DMAP (3 mg, 0.026 mmol), TEA (68 µL, 0.49 mmol) and Ac_2O (47 µL, 0.49 mmol, 98%) were added at room temperature to a stirring solution of the above crude alcohol in anhydrous CH_2Cl_2 (1.0 mL). After 18 h, saturated aqueous $NaHCO_3$ was added and the resulting mixture was extracted with CH_2Cl_2. The combined organic extracts were dried and concentrated. Flash chromatography (from hexane to 95:5 hexane–Et_2O) afforded compound **19** (123 mg, 57%) as a colorless oil: $[\alpha]^{20}_{D}$ = +52.84 (*c* 0.66, $CHCl_3$); IR (NaCl): 1691, 1738 cm^{-1}; ^{1}H-NMR (500MHz, $CDCl_3$, COSY, *g*-HSQC) *δ*: 5.88-5.80 (m, 1H, *H*C=CH_2), 5.22 (t, *J* = 7.5 Hz, 1H, H-4), 5.03 (dd, *J* = 1.5, 16.8 Hz, 1H, HC=C*H_2*), 4.94 (dd, *J* = 1.5, 10.4 Hz, 1H, HC=C*H_2*), 4.02-4.00 (m, 1H, H-2), 2.48-2.35 (m, 2H), 2.26-2.24 (m, 1H, H-4a), 2.18 (*ddd*, *J* = 6.5, 7.5, 16.0 Hz, 1H, H-3), 2.04 (s, 3H, CH_3C=O), 1.98-1.90 (m, 3H), 1.88 (dd, *J* = 2.0, 16.0 Hz, 1H, H-3), 1.66-1.58 (m, 3H), 1.45 [s, 9H, (OC(CH_3)$_3$], 1.53-1.38 (m, 5H), 1.36-1.21 (m, 5H), 0.90 (t, *J* = 7.0 Hz, 3H, CH_3); ^{13}C-NMR (100.6 MHz, $CDCl_3$) *δ*: 170.9 (C=O), 154.8 (NCO), 138.9

(H*C*=CH_2), 114.3 (HC=*C*H_2), 79.3 [O*C*(CH_3)$_3$], 69.0 (C-4), 59.3 (C-8a), 51.0 (C-2), 37.3 (C-4a), 36.2 (CH_2), 33.5 (CH_2), 32.0 (CH_2), 30.4 (CH_2), 29.5 (CH_2), 28.6 (CH_2), 28.5 [OC(*C*H_3)$_3$], 24.4 (CH_2), 22.6 (CH_2), 21.5 (CH_2), 21.3 (*C*H_3C=O), 19.6 (CH_2), 14.1 (CH_3); HRMS (ESI-TOF) m/z: $[M + H]^+$ Calcd for $C_{24}H_{42}NO_4$ 408.3108; Found 408.3107.

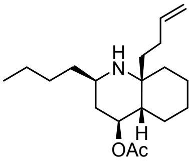

(2*R*,4*S*,4a*S*,8a*S*)-4-Acetoxy-8a-(3-butenyl)-2-butyldecahydroquinoline (20): TFA (23 µL, 0.3 mmol) was added to stirring solution of **19** (12 mg, 0.03 mmol) in anhydrous CH_2Cl_2 (290 µL) and the mixture was stirred for 1 h at room temperature. Then, the solvent was evaporated, the residue was dissolved in CH_2Cl_2, and the solution was washed with 5% aqueous NaOH, dried and concentrated. Flash chromatography (KP-NH Biotage® SNAP cartridge, 9:1 hexane–EtOAc), afforded compound **20** (8 mg, 89%) as a colorless oil: $[\alpha]^{20}_D$ = + 41.3 (*c* 0.35, $CHCl_3$); IR (NaCl): 1739 cm^{-1}; ^{1}H-NMR (500 MHz, $CDCl_3$, COSY, *g*-HSQC) δ: 5.91-5.83 (m, 1H, C*H*=CH_2), 5.20 (td, *J* = 4.8, 11.5 Hz, 1H, H-4), 5.07 (d, *J* = 17.0 Hz, 1H, CH=C*H*$_2$), 4.98 (d, *J* = 10.0 Hz, 1H, CH=C*H*$_2$), 2.97 (br s, 1H, H-2), 2.21-2.08 (m, 3H, C-4a), 2.05 (s, 3H, $COCH_3$), 1.85 (td, *J* = 3.5, 12.5 Hz, 3H), 1.67-1.49 (m, 6H), 1.46-1.25 (m, 9H), 0.88 (t, *J* = 6.7 Hz, 3H, CH_3); Several of the signals in the ^{13}C-NMR spectrum at 25 °C were broad and ill-defined, thus indicating the existence of a slow conformational equilibrium. ^{13}C-NMR (125 MHz, $CDCl_3$) δ: 170.8 (C=O), 139.0 (*C*H=CH_2), 114.9 (CH=*C*H_2), 69.7 (C-4), 48.2 (C-8a), 43.3 (C-2), 37.3 (C-4a), 35.0 (CH_2), 31.9 (CH_2), 29.7 (CH_2), 27.9 (CH_2), 26.7 (CH_2), 22.6 (CH_2), 22.4 (CH_2), 22,3 (CH_2), 22.1 (CH_2), 21.2 (CH_2), 19.5 (CH_2), 14.0 (CH_2); HRMS (ESI-TOF) m/z: $[M + H]^+$ Calcd for $C_{19}H_{34}NO_2$ 308.2584; Found 308.2580.

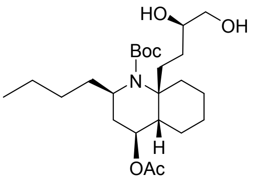

(2*R*,4*S*,4a*S*,8a*S*)-4-Acetoxy-8a-[(*R*)-3,4-dihydroxybutyl]-1-(*tert*-butoxycarbonyl)-2-butyldecahydroquinoline (21): A solution of **19** (45 mg, 0.11 mmol) in 1:1 *tert*-BuOH-H_2O (1.9 mL) was added at 0°C to a stirred solution of AD-mix-β (287 mg) in 1:1 *tert*-BuOH-H_2O (1 mL), and the resulting mixture was stirred for 4 h at this temperature. EtOAc (2.9 mL) and $Na_2S_2O_3$ (287 mg) were added, and the resulting mixture was extracted with EtOAc. The combined organic extracts were dried and concentrated. Flash chromatography (from 100 to 98:2 CH_2Cl_2–MeOH) afforded compound **21** (45 mg, 92%; 78:22 mixture of C-3’ epimers, calculated by GC/MS) as a colorless oil: IR (NaCl): 1670, 1738, 3489 cm^{-1}; ^{1}H-NMR (400 MHz, $CDCl_3$, COSY, *g*-HSQC) δ: 5.19 (t, J = 8.0 Hz, 1H, H-4), 4.01-3.95 (m, 1H, H-2), 3.73-3.68 (m, 1H, H-3’), 3.64-3.61 (m, 1H, H-4’), 3.52-3.43 (m, 1H, H-4’), 2.55-2.33 (m, 3H), 2.21-2.14 (m, 2H), 2.03 (s, 3H, CH_3CO), 1.99-1.92 (m, 1H), 1.86 (dd, J = 1.6, 16.0 Hz, 1H), 1.73-1.67 (m, 1H), 1.63-1.57 (m, 2H), 1.43 [s, 9H, $OC(CH_3)_3$], 1.55-1.38 (m, 5H) 1.35-1.19 (m, 6H), 0.89 (t, J = 7.2 Hz, 3H, CH_3); ^{13}C-NMR (100.6 MHz, $CDCl_3$) δ: 171.0 (C=O), 154.8 (NCO), 79.6 and 79.4 [O*C*(CH_3)$_3$], 72.5 and 72.0 (C-4), 68.8 (C-3’), 66.7 (C-4’), 59.6 and 59.5 (C-8a), 51.1 (C-2), 37.3 and 37.1 (C-4a), 36.1 and 36.0 (CH_2), 33.4 and 33.3 (CH_2), 30.3 and 30.2 (CH_2), 29.4 (CH_2), 28.6 [OC(*C*H_3)$_3$], 27.8 (CH_2), 27.5 (CH_2), 24.4 (CH_2), 22.5 (CH_2), 21.4 (CH_2), 21.3 (*C*H_3CO), 19.5 (CH_2), 14.1 (CH_3); HRMS (ESI-TOF) m/z: $[M + H]^+$ Calcd for $C_{24}H_{44}NO_6$ 442.3163; Found 442.3174.

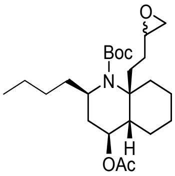

(2*R*,4*S*,4a*S*,8a*S*)-4-Acetoxy-1-(*tert*-Butoxycarbonyl)-2-butyl-8a-{[(*R*)-oxiran-2-yl]ethyl}decahydroquinoline (22): *1st step*: 2,4,6-collidine (34 µL, 0.255 mmol) and MsCl (10 µL, 0.122 mmol) were added at –78 °C to a stirred solution of diol **21** (45 mg, 0.102 mmol) in anhydrous CH_2Cl_2 (2.6 mL), and the mixture was stirred at this temperature for 3 h. Then, the mixture was warmed to room temperature and the stirring was continued for 17 h. The mixture was diluted with CH_2Cl_2 and washed with water. The combined organic extracts were dried and concentrated to give a crude mesylate.

2nd step: NaH (5 mg, 0.18 mmol) was added at 0°C to a solution of the above mesylate in anhydrous THF (350 µL), and the mixture was stirred at room temperature for 1 h 15 min. H_2O was added and the resulting mixture was extracted with Et_2O. The combined organic extracts were washed with brine, dried, and concentrated. Flash chromatography (from 9:1 to 8:2 hexane–EtOAc) afforded epoxide **22** as a colorless oil (mixture of C-3' epimers; 28 mg, 64%): ^{1}H-NMR (400 MHz, $CDCl_3$) δ: 5.20 (t, J = 8.0 Hz, 1H, H-4), 4.03-3.96- (br s, 1H), 2.96-2.88 (m, 1H), 2.78-2.74 (m, 1H), 2.53-2.46 (m, 3H), 2.20-2.10 (m, 2H), 2.04 (s, 3H, CH_3C=O), 1.96-1.84 (m, 2H), 1.62-1.56 (m, 4H), 1.45 [s, 9H, $OC(CH_3)_3$], 1.55-1.38 (m, 3H), 1.37-1.28 (m, 4H), 1.27-1.20 (m, 4H), 0.89 (t, J = 7.0 Hz, 3H, CH_3); ^{13}C-NMR (100.6 MHz, $CDCl_3$) δ: 170.8 (C=O), 154.7 and 154.5 (NCO), 79.6 and 79.2 [O*C*(CH_3)$_3$], 66.8 and 68.7 (C-4), 59.3 and 59.0 (C-8a), 52.6 and 52.3 (C-2), 50.9 (C-3'), 47.4 and 46.8 (C-4'), 37.3 and 37.1 (C-4a), 36.1 and 36.0 (CH_2), 33.4 and 33.3 (CH_2), 30.3 and 30.2 (CH_2), 29.4 (CH_2), 28.5 and 28.4 [OC(*C*H_3)$_3$], 28.0 and 27.9 (CH_2), 26.9 (CH_2), 24.2 and 23.9 (CH_2), 22.5 and 22.4 (CH_2), 21.5 and

21.4 (CH_2), 21.3 (CH_3CO), 19.5 (CH_2), 14.1 (CH_3); HRMS (ESI-TOF) m/z: $[M + H]^+$ Calcd for $C_{24}H_{42}NO_5$ 424.3057; Found 424.3049.

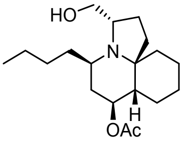

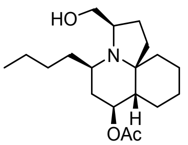

(3*S*,5*R*,7*S*,7a*S*,11a*S*)-7-Acetoxy-5-butyl-3-(hydroxymethyl)perhydropyrrolo[2,1-*j*]quinoline (23) and (3*R*,5*R*,7*S*,7a*S*,11a*S*)-7-acetoxy-5-butyl-3-(hydroxymethyl)-perhydropyrrolo[2,1-*j*]quinoline (*epi*-23): TFA (27 µL, 0.36 mmol) was added to a stirred solution of **22** (28 mg, 0.066 mmol) in CH_2Cl_2 (1.7 mL). After 45 min, the mixture was cooled to 0 °C and 15% aqueous KOH was added. The aqueous phase was extracted with CH_2Cl_2 and the combined organic extracts were dried and concentrated. Flash chromatography (hexane to 9:1 hexane–EtOAc), afforded **23** (12 mg, 57%) and **3-*epi*-23** (3 mg, 14%) as colorless oils. **3-*epi*-23** (higher R_f): ^{1}H-NMR (400 MHz, $CDCl_3$) δ: 5.34-5.27 (m, 1H), 3.56 (d, *J* = 9.6 Hz, 1H), 3.35 (d, *J* = 9.6 Hz, 1H), 3.20-3.18 (m, 1H), 2.90 (br s, 1H), 2.18-2.09 (m, 4H), 2.05 (s, 3H, $COCH_3$), 1.87-1.62 (m, 7H), 1.59-1.52 (m, 2H), 1.49-1.40 (m, 4H), 1.21-1.07 (m, 5H), 0.88 (br s, 3H, CH_3); HRMS (ESI-TOF) m/z: $[M + H]^+$ Calcd for $C_{19}H_{34}NO_3$ 324.2533; Found 324.2531. **23** (lower R_f): $[\alpha]^{20}_D$ = +29.7 (c 0.40, CH_2Cl_2); IR (NaCl): 1738, 3510 cm^{-1}; ^{1}H-NMR (500 MHz, $CDCl_3$) δ: 5.14 (td, *J* = 4.5, 11.0 Hz, 1H, H-4), 3.49-3.45 (m, 1H), 3.36-3.34 (m, 2H), 3.05 (br s, 1H), 2.21-2.12 (m, 4H), 2.05 (s, 3H, $COCH_3$), 1.94 (td, *J* = 4.5, 13.0 Hz, 1H), 1.86-1.81 (m, 1H), 1.73-1.65 (m, 4H), 1.59-1.51 (m, 3H), 1.43-1.27 (m, 9H), 0.89 (t, *J* = 6.7 Hz, 3H, CH_3); ^{13}C-NMR (125 MHz, $CDCl_3$,) δ: 170.7 (COO), 70.9 (C-4), 67.5 (C-8a), 66.4 (CH), 56.3 (C-13), 52.6 (C-2), 39.6 (C-4a), 36.5 (CH_2), 34.5 (CH_2), 33.9 (CH_2), 31.7 (CH_2), 29.7 (CH_2), 28.9 (CH_2), 24.5 (CH_2), 23.7 (CH_2), 22.7 (CH_2), 21.2 ($COCH_3$),

20.5 (CH_2), 14.1 (CH_3); HRMS (ESI-TOF) m/z: $[M + H]^+$ Calcd for $C_{19}H_{34}NO_3$ 324.2533; Found 324.2529.

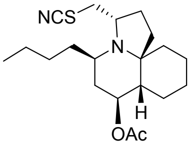

(–)-**Cylindricine H:** DEAD (19 mg, 0.11 mmol) and PPh_3 (29 mg, 0.11 mmol) were sequentially added to a stirred solution of NH_4SCN (11 mg, 0.138 mmol) in anhydrous CH_2Cl_2 (0.6 mL). After 30 min, a solution of **23** (6 mg, 0.018 mmol) in anhydrous CH_2Cl_2 (1.6 mL) was added and the resulting mixture was stirred for 1 h at room temperature. Saturated aqueous $NaHCO_3$ was added, and the aqueous phase was separated and extracted with Et_2O. The combined organic extracts were dried and concentrated. Flash chromatography (15:1 hexane–EtOAc) afforded (–)-**cylindricine H** as a colorless oil (6.3 mg, 94%). $[\alpha]^{20}_{D}$ = –8.5 (*c* 0.47, MeOH); IR (NaCl): 1732, 2083, 2154 cm^{-1}; ^{1}H-NMR (500 MHz, $CDCl_3$) δ: 5.11-5.01 (m, 1H), 3.60-3.56 (m, 1H), 3.20-3.15 (m, 1H), 3.08-3.04 (m, 1H), 3.00-2.92 (m, 1H), 2.27-2.20 (m, 1H), 2.14-2.07 (m, 2H), 2.02 (s, 3H, $COCH_3$), 1.87-1.80 (m, 2H), 1.74-1.51 (m, 5H), 1.43-1.20 (m, 11H), 0.91 (t, J = 8 Hz, 3H, CH_3); ^{13}C-NMR (100.6 MHz, $CDCl_3$) δ: 170.7, 114.2, 70.8, 67.4, 54.3, 52.3, 45.0, 40.1, 35.7, 34.5, 33.3, 31.8, 29.5, 29.2, 24.5, 23.8, 22.8, 21.2, 20.4, 14.0; HRMS (ESI-TOF) m/z: $[M + H]^+$ Calcd for $C_{20}H_{33}N_2O_2S$ 365.2257; Found 365.2262.

II) Comparison of the 13C NMR of natural and synthetic Cylindricine H

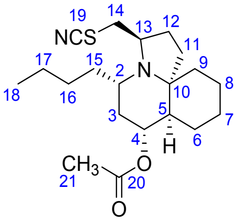

Position	Natural δ_C	Our sample δ_C	$\Delta\delta_C$ (ppm)
18	14.7	14.0	0.7
8	21.1*	20.4	0.7
21	21.9	21.2	0.7
6	23.5*	22.8	0.7
7	24.4*	23.8	0.6
17	25.2*	24.5	0.7
12	29.8	29.2	0.6
16	30.1*	29.5	0.6
3	32.5	31.8	0.7
15	34.0	33.3	0.7
9	35.1*	34.5	0.6
11	36.4*	35.7	0.7
5	40.8	40.1	0.7
14	45.6	45.0	0.6
2	52.9	52.3	0.6
13	55.0	54.3	0.7
10	68.0	67.4	0.6
4	71.4	70.8	0.6
19	114.8	114.2	0.6
20	171.3	170.7	0.6

*Assignments are tentative

III) Copies of ^{1}H and ^{13}C NMR spectra

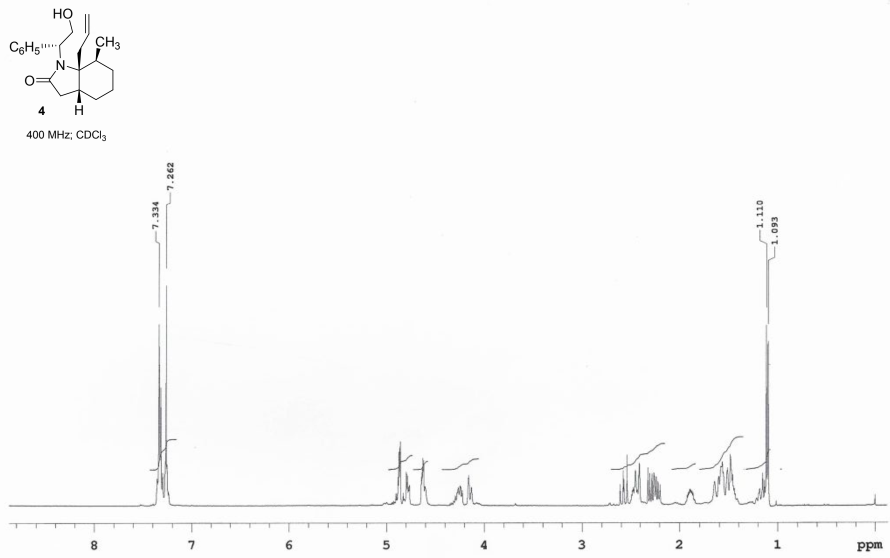
HO
C6H5
N
CH3
O
H
4
400 MHz; CDCl3
7.334
7.262
1.110
1.093
8
7
6
5
4
3
2
1
ppm

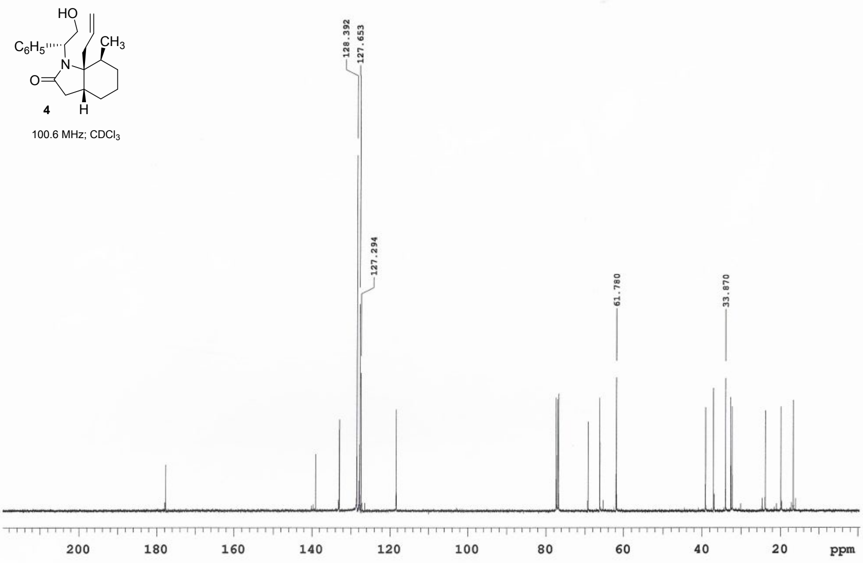
HO
C6H5
N
CH3
O
H
4
100.6 MHz; CDCl3
128.392
127.653
127.294
61.780
33.870
200
180
160
140
120
100
80
60
40
20
ppm

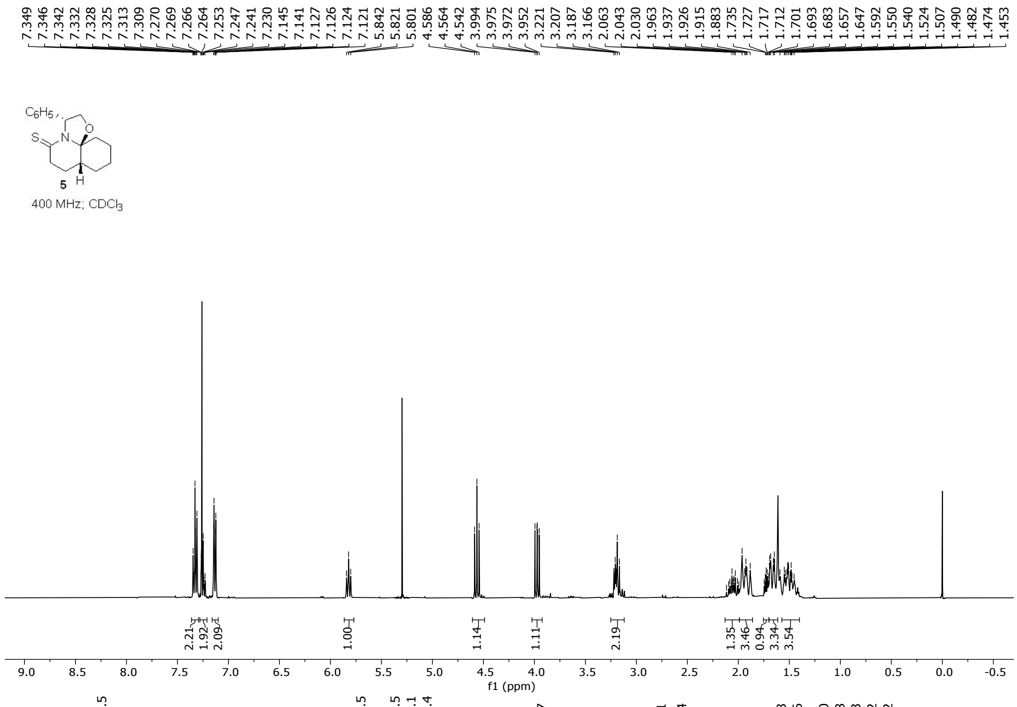

C6H5
S
N
O
H
5
400 MHz; CDCl3
f1 (ppm)

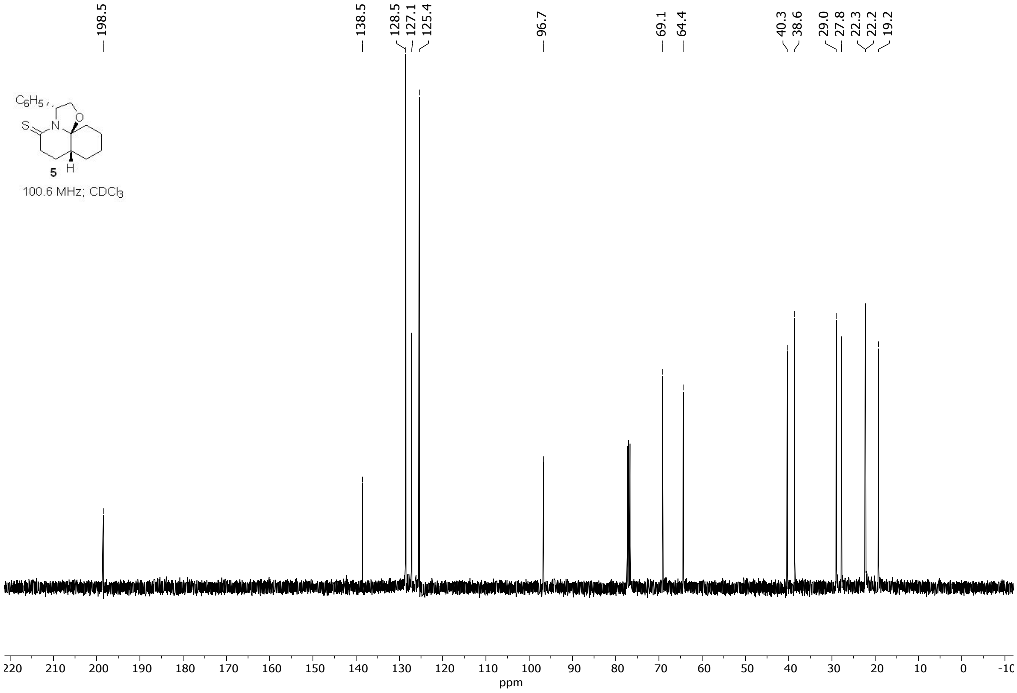

198.5
138.5
128.5
127.1
125.4
96.7
69.1
64.4
40.3
38.6
29.0
27.8
22.3
22.2
19.2
C6H5
S
N
O
H
5
100.6 MHz; CDCl3
ppm

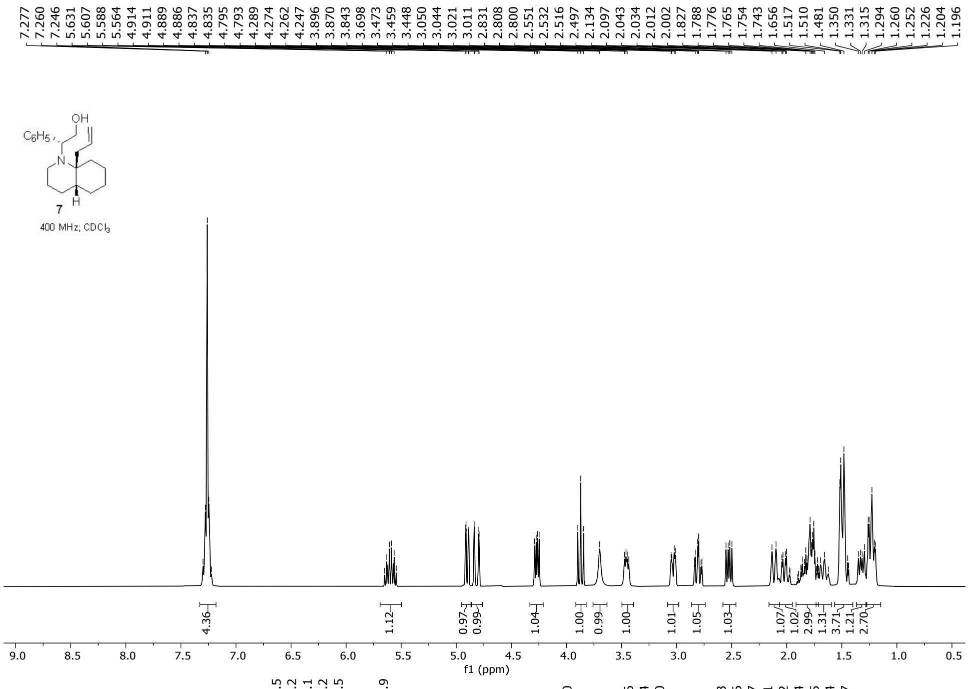
7.277
7.260
7.246
5.631
5.607
5.588
5.564
4.914
4.911
4.889
4.886
4.837
4.835
4.795
4.793
4.289
4.274
4.262
4.247
3.896
3.870
3.843
3.698
3.473
3.459
3.448
3.050
3.044
3.021
3.011
2.831
2.808
2.800
2.551
2.532
2.516
2.497
2.134
2.097
2.043
2.034
2.012
2.002
1.827
1.788
1.776
1.765
1.754
1.743
1.656
1.517
1.510
1.481
1.350
1.331
1.315
1.294
1.260
1.252
1.226
1.204
1.196
OH
C_6H_5
N
H
7
400 MHz; $CDCl_3$
4.36
1.12
0.97
0.99
1.04
1.00
0.99
1.00
1.01
1.05
1.03
1.07
1.02
2.99
1.31
3.71
1.21
2.70
f1 (ppm)

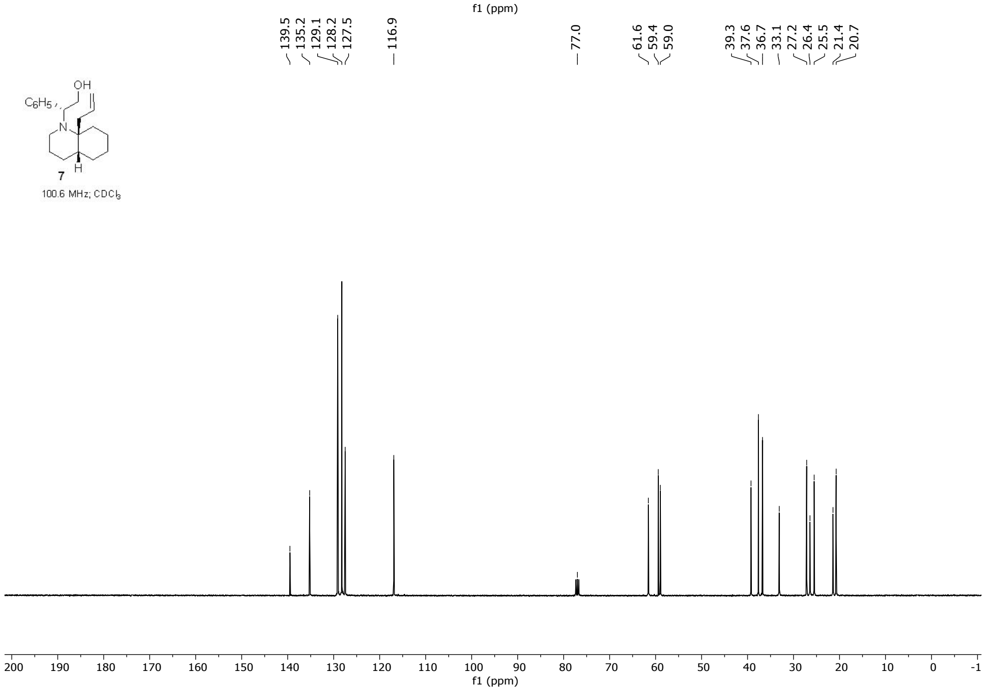
139.5
135.2
129.1
128.2
127.5
116.9
77.0
61.6
59.4
59.0
39.3
37.6
36.7
33.1
27.2
26.4
25.5
21.4
20.7
OH
C_6H_5
N
H
7
100.6 MHz; $CDCl_3$
f1 (ppm)

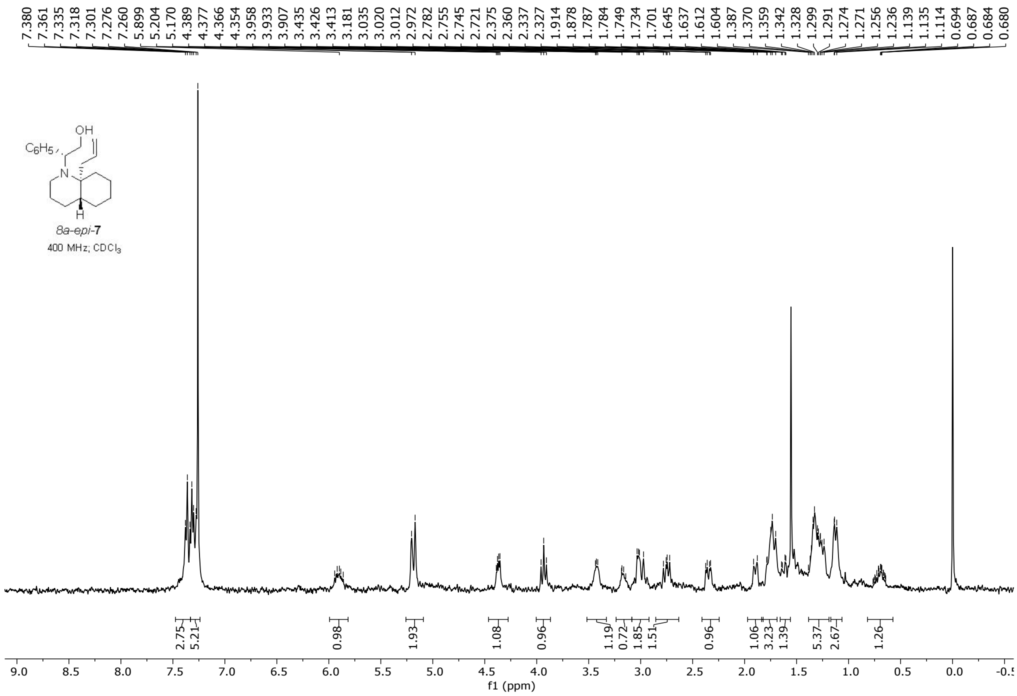
7.380 7.361 7.335 7.318 7.301 7.276 7.260 5.899 5.204 5.170 4.389 4.377 4.366 4.354 3.958 3.933 3.907 3.435 3.426 3.413 3.181 3.035 3.020 3.012 2.972 2.782 2.755 2.745 2.721 2.375 2.360 2.337 2.327 1.914 1.878 1.787 1.784 1.749 1.734 1.701 1.645 1.637 1.612 1.604 1.387 1.370 1.359 1.342 1.328 1.299 1.291 1.274 1.271 1.256 1.236 1.139 1.135 1.114 0.694 0.687 0.684 0.680
OH
C6H5
N
H
8a-epi-7
400 MHz; CDCl3
2.75 5.21 0.98 1.93 1.08 0.96 1.19 0.72 1.85 1.51 0.96 1.06 3.23 1.39 5.37 2.67 1.26
f1 (ppm)

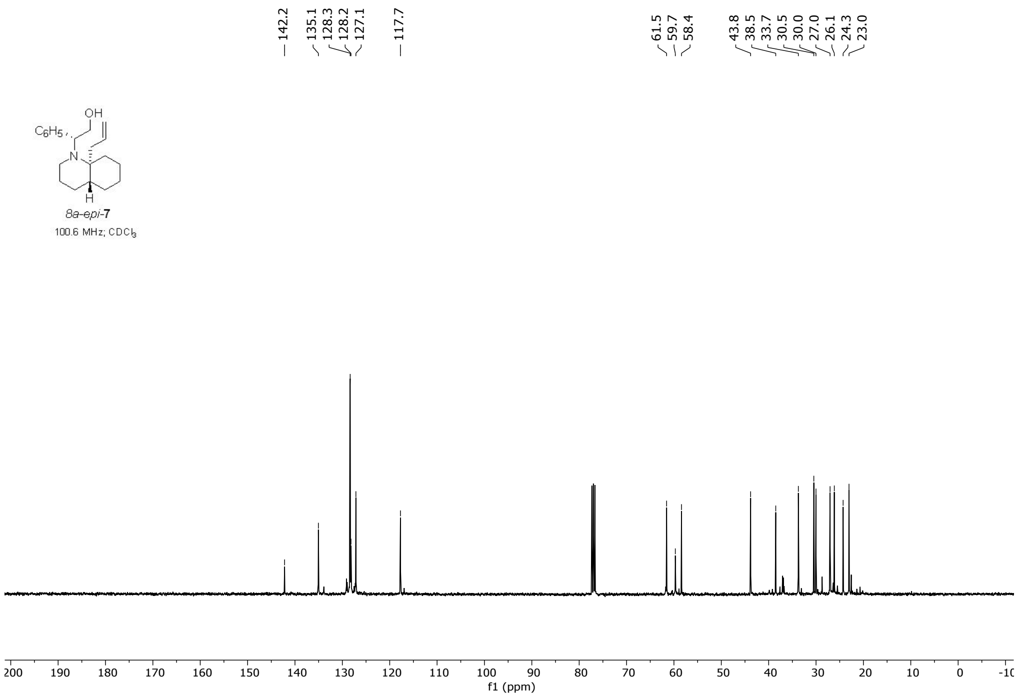
142.2 135.1 128.3 128.2 127.1 117.7 61.5 59.7 58.4 43.8 38.5 33.7 30.5 30.0 27.0 26.1 24.3 23.0
OH
C6H5
N
H
8a-epi-7
100.6 MHz; CDCl3
f1 (ppm)

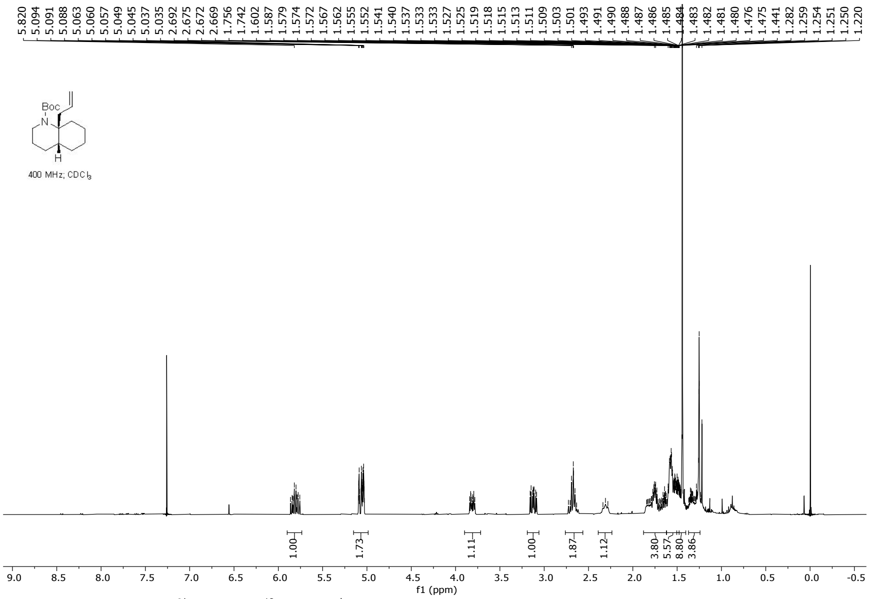
5.820 5.094 5.091 5.088 5.063 5.060 5.057 5.049 5.045 5.037 5.035 2.692 2.675 2.672 2.669 1.756 1.742 1.602 1.587 1.579 1.574 1.572 1.567 1.562 1.555 1.552 1.541 1.540 1.537 1.533 1.533 1.527 1.525 1.519 1.518 1.515 1.513 1.511 1.509 1.503 1.501 1.493 1.491 1.490 1.488 1.487 1.486 1.485 1.484 1.483 1.482 1.481 1.480 1.476 1.475 1.441 1.282 1.259 1.254 1.251 1.250 1.220
Boc
N
H
400 MHz; CDCl3
1.00 1.73 1.11 1.00 1.87 1.12 3.80 5.57 8.80 3.86
f1 (ppm)

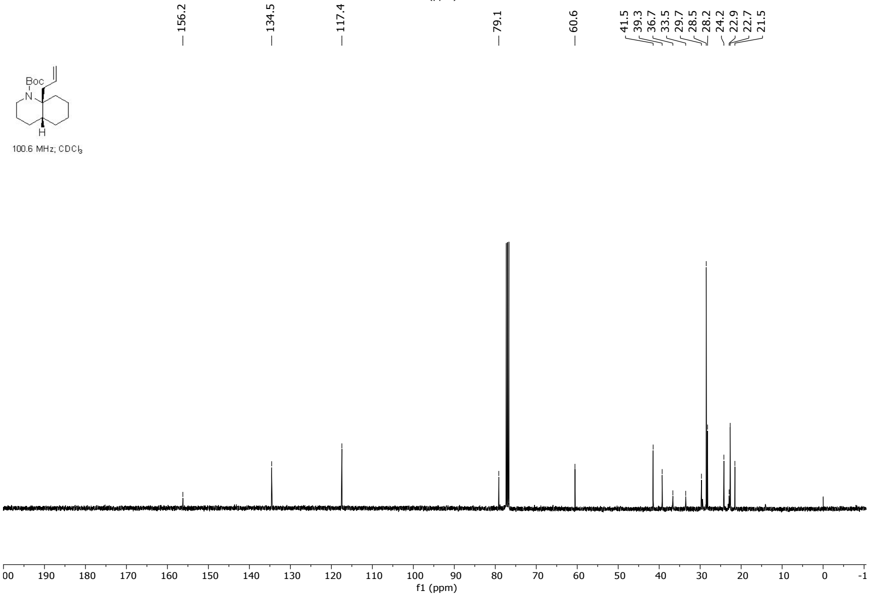
156.2 134.5 117.4 79.1 60.6 41.5 39.3 36.7 33.5 29.7 28.5 28.2 24.2 22.9 22.7 21.5
Boc
N
H
100.6 MHz; CDCl3
f1 (ppm)

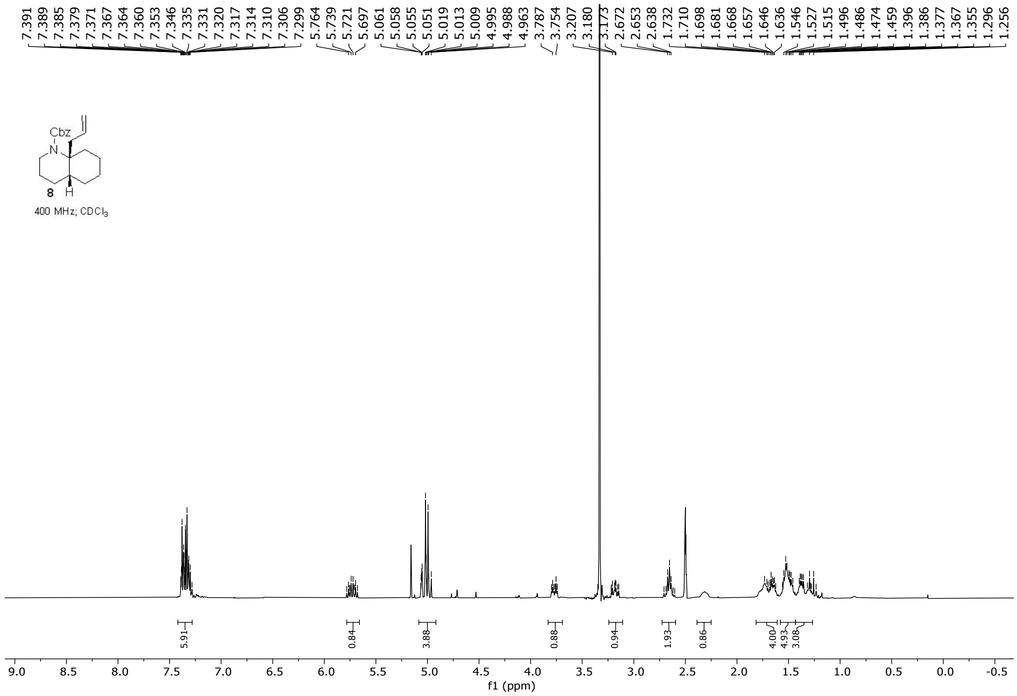
7.391
7.389
7.385
7.379
7.371
7.367
7.364
7.360
7.353
7.346
7.335
7.331
7.320
7.317
7.314
7.310
7.306
7.299
5.764
5.739
5.721
5.697
5.061
5.058
5.055
5.051
5.019
5.013
5.009
4.995
4.988
4.963
3.787
3.754
3.207
3.180
3.173
2.672
2.653
2.638
1.732
1.710
1.698
1.681
1.668
1.657
1.646
1.636
1.546
1.527
1.515
1.496
1.486
1.474
1.459
1.396
1.386
1.377
1.367
1.355
1.296
1.256
Cbz
N
8
H
400 MHz; CDCl3
5.91
0.84
3.88
0.88
0.94
1.93
0.86
4.00
4.93
3.08
9.0
8.5
8.0
7.5
7.0
6.5
6.0
5.5
5.0
4.5
4.0
3.5
3.0
2.5
2.0
1.5
1.0
0.5
0.0
-0.5
f1 (ppm)

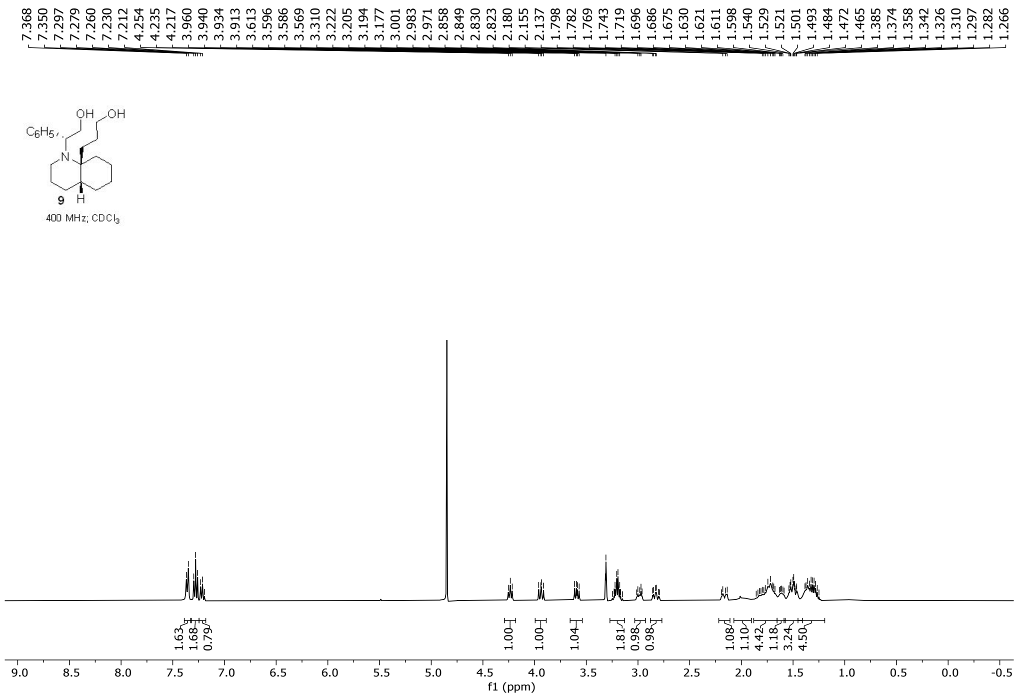
7.368 7.350 7.297 7.279 7.260 7.230 7.212 4.254 4.235 4.217 3.960 3.940 3.934 3.913 3.613 3.596 3.586 3.569 3.310 3.222 3.205 3.194 3.177 3.001 2.983 2.971 2.858 2.849 2.830 2.823 2.180 2.155 2.137 1.798 1.782 1.769 1.743 1.719 1.696 1.686 1.675 1.630 1.621 1.611 1.598 1.540 1.529 1.521 1.501 1.493 1.484 1.472 1.465 1.385 1.374 1.358 1.342 1.326 1.310 1.297 1.282 1.266
OH
OH
C6H5
N
9
H
400 MHz; CDCl3
1.63 1.68 0.79 1.00 1.00 1.04 1.81 0.98 0.98 1.08 1.10 4.42 1.18 3.24 4.50
9.0 8.5 8.0 7.5 7.0 6.5 6.0 5.5 5.0 4.5 4.0 3.5 3.0 2.5 2.0 1.5 1.0 0.5 0.0 -0.5
f1 (ppm)

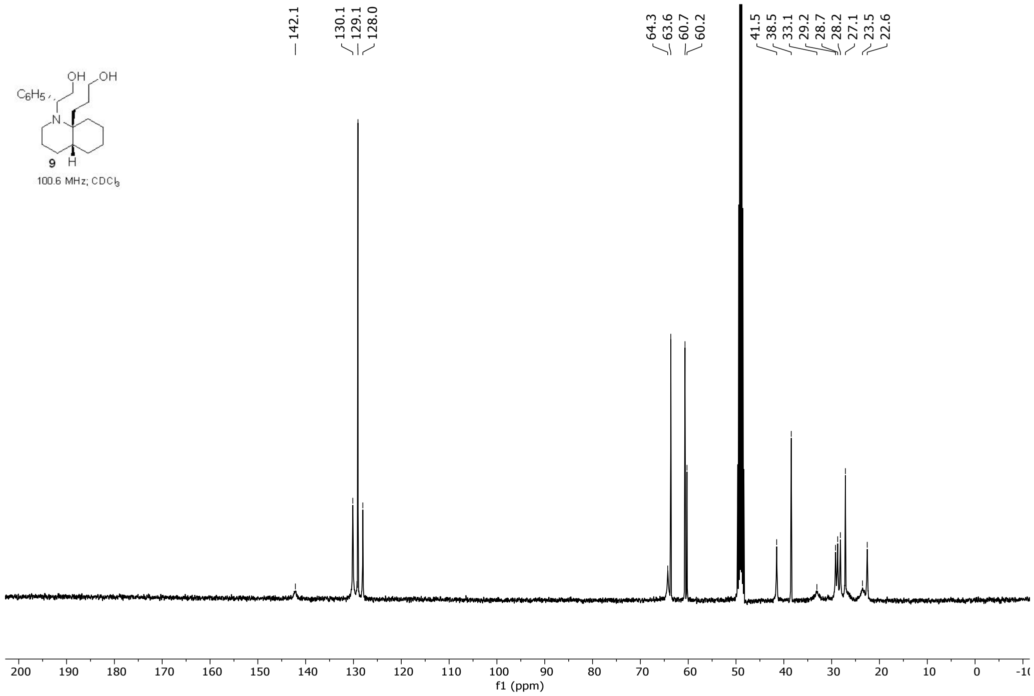
142.1
130.1 129.1 128.0
64.3 63.6 60.7 60.2
41.5 38.5 33.1 29.2 28.7 28.2 27.1 23.5 22.6
OH
OH
C6H5
N
9
H
100.6 MHz; CDCl3
200 190 180 170 160 150 140 130 120 110 100 90 80 70 60 50 40 30 20 10 0
f1 (ppm)

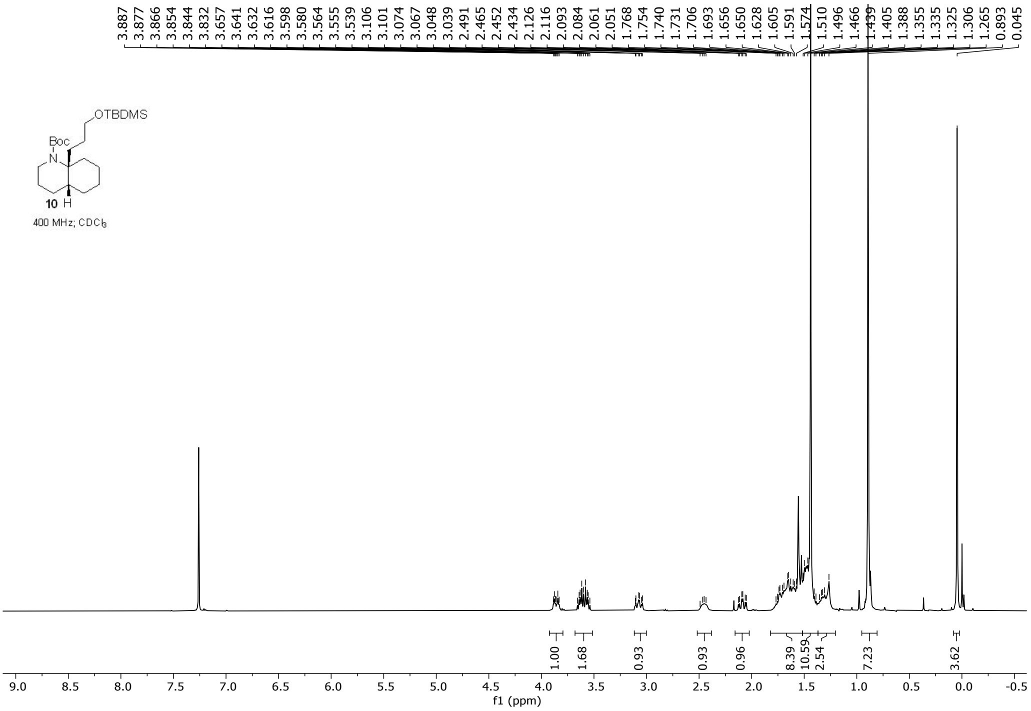

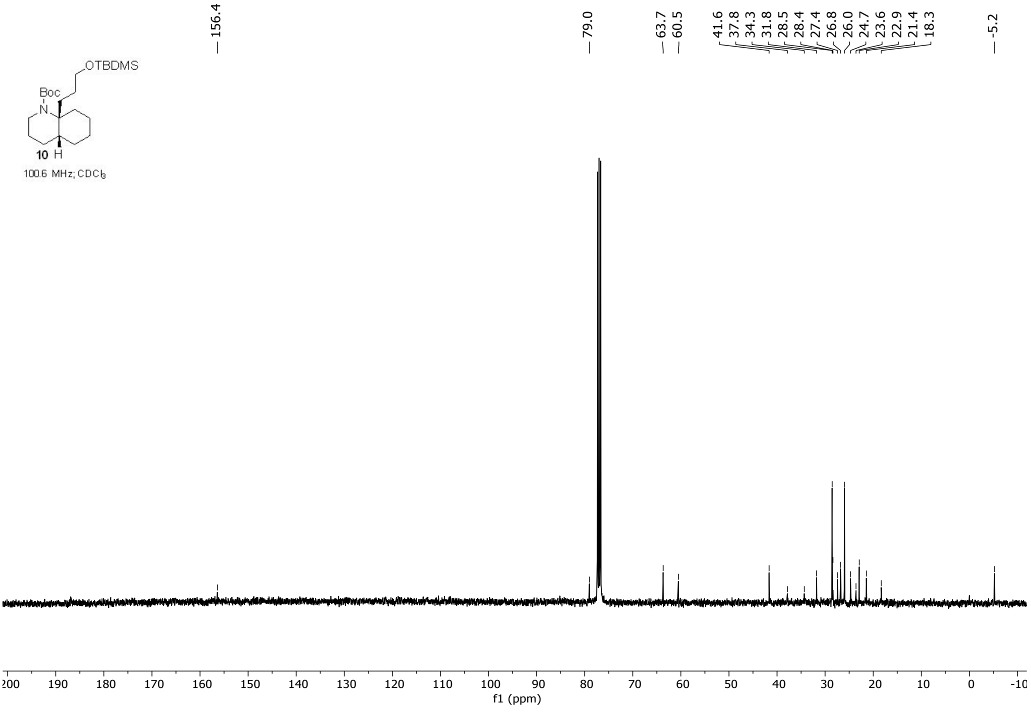

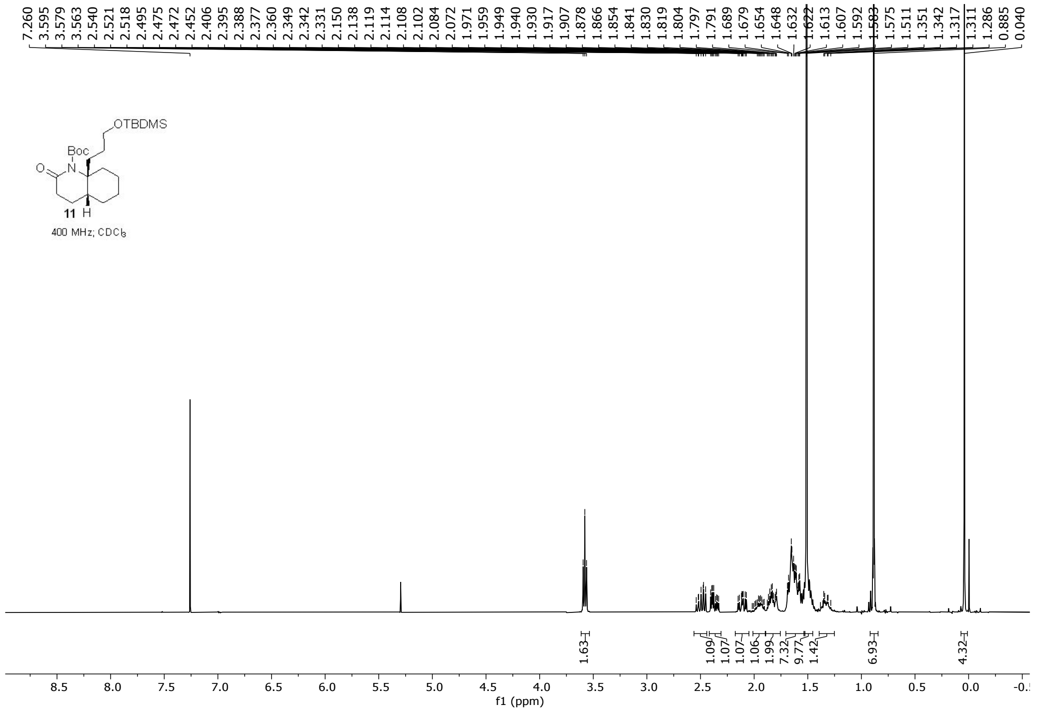

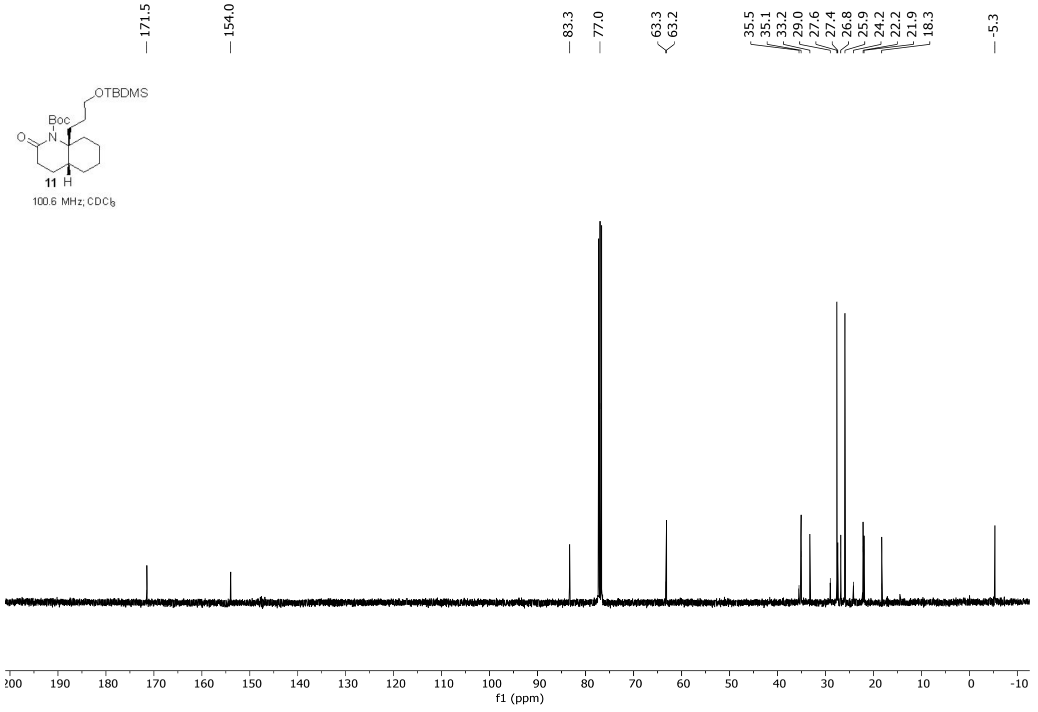

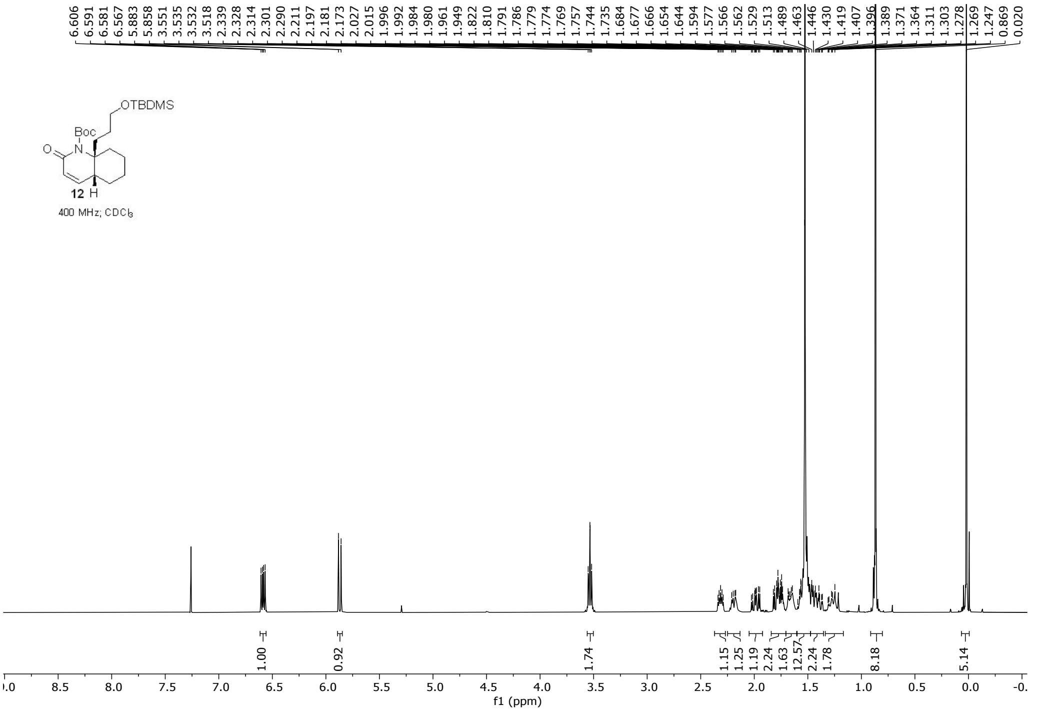
6.606 6.591 6.581 6.567 5.883 5.858 3.551 3.535 3.532 3.518 2.339 2.328 2.314 2.301 2.290 2.211 2.197 2.181 2.173 2.027 2.015 1.996 1.992 1.984 1.980 1.961 1.949 1.822 1.810 1.791 1.786 1.779 1.774 1.769 1.757 1.744 1.735 1.684 1.677 1.666 1.654 1.644 1.594 1.577 1.566 1.562 1.529 1.513 1.489 1.463 1.446 1.430 1.419 1.407 1.396 1.389 1.371 1.364 1.311 1.303 1.278 1.269 1.247 0.869 0.020
OTBDMS
Boc
O
N
12 H
400 MHz; CDCl3
1.00
0.92
1.74
1.15
1.25
1.19
2.24
1.63
12.57
2.24
1.78
8.18
5.14
f1 (ppm)

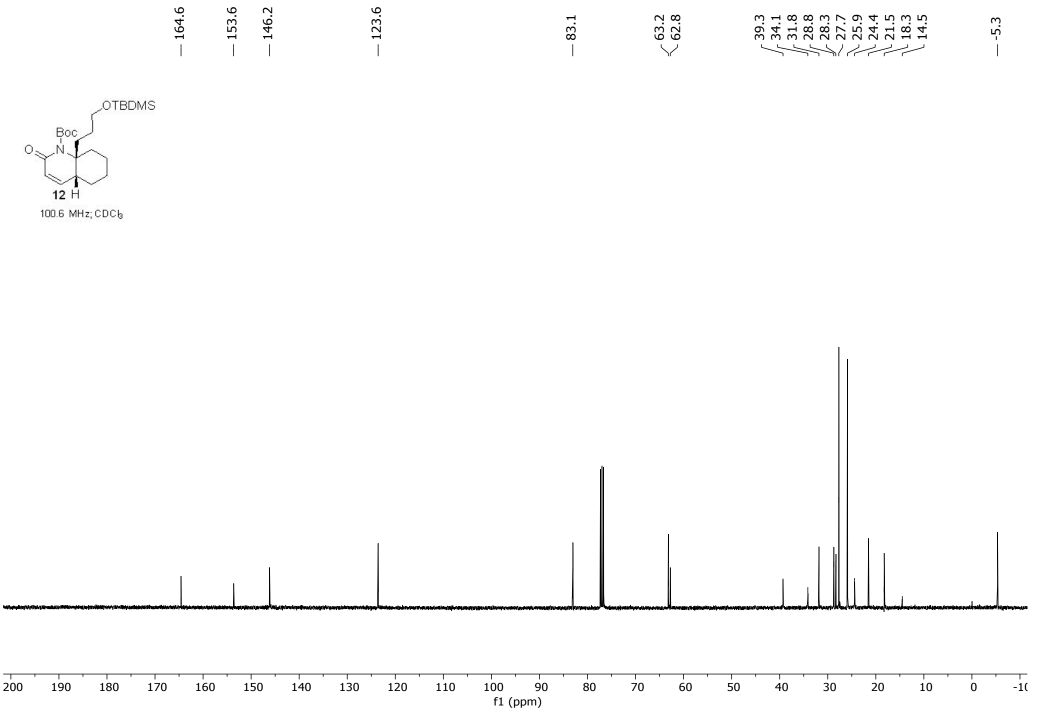
164.6
153.6
146.2
123.6
83.1
63.2
62.8
39.3 34.1 31.8 28.8 28.3 27.7 25.9 24.4 21.5 18.3 14.5
-5.3
OTBDMS
Boc
O
N
12 H
100.6 MHz; CDCl3
f1 (ppm)

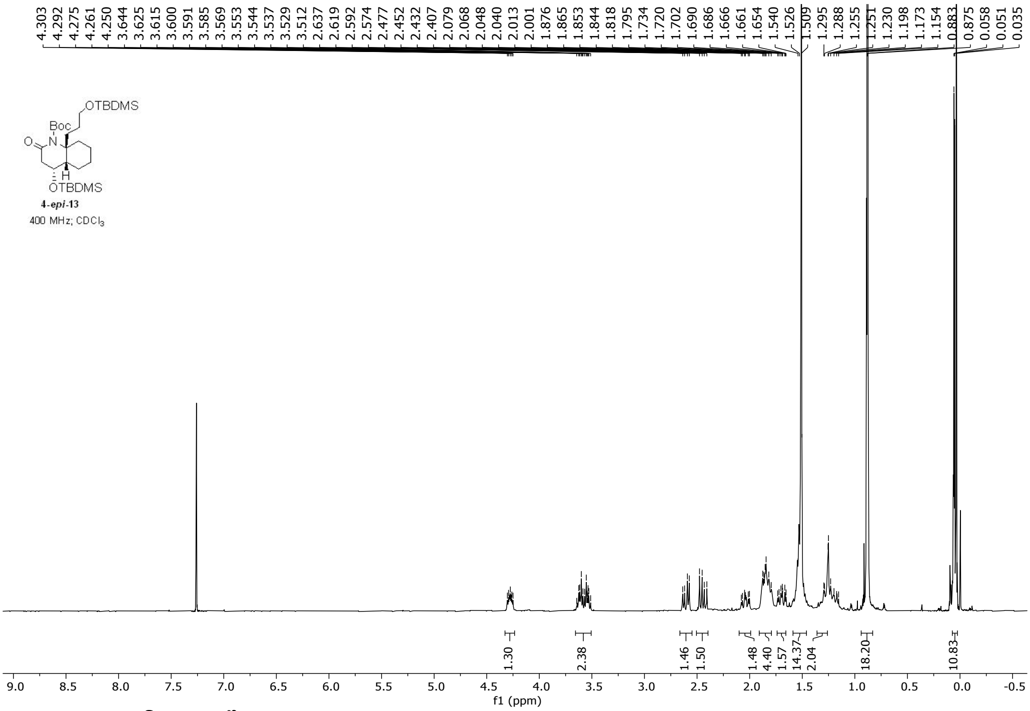
4.303
4.292
4.275
4.261
4.250
3.644
3.625
3.615
3.600
3.591
3.585
3.569
3.553
3.544
3.537
3.529
3.512
2.637
2.619
2.592
2.574
2.477
2.452
2.432
2.407
2.079
2.068
2.048
2.040
2.013
2.001
1.876
1.865
1.853
1.844
1.818
1.795
1.734
1.720
1.702
1.690
1.686
1.666
1.661
1.654
1.540
1.526
1.509
1.295
1.288
1.255
1.251
1.230
1.198
1.173
1.154
0.883
0.875
0.058
0.051
0.035
OTBDMS
Boc
O
N
H
OTBDMS
4-epi-13
400 MHz; CDCl3
1.30
2.38
1.46
1.50
1.48
4.40
1.57
14.37
2.04
18.20
10.83
9.0
8.5
8.0
7.5
7.0
6.5
6.0
5.5
5.0
4.5
4.0
3.5
3.0
2.5
2.0
1.5
1.0
0.5
0.0
-0.5
f1 (ppm)

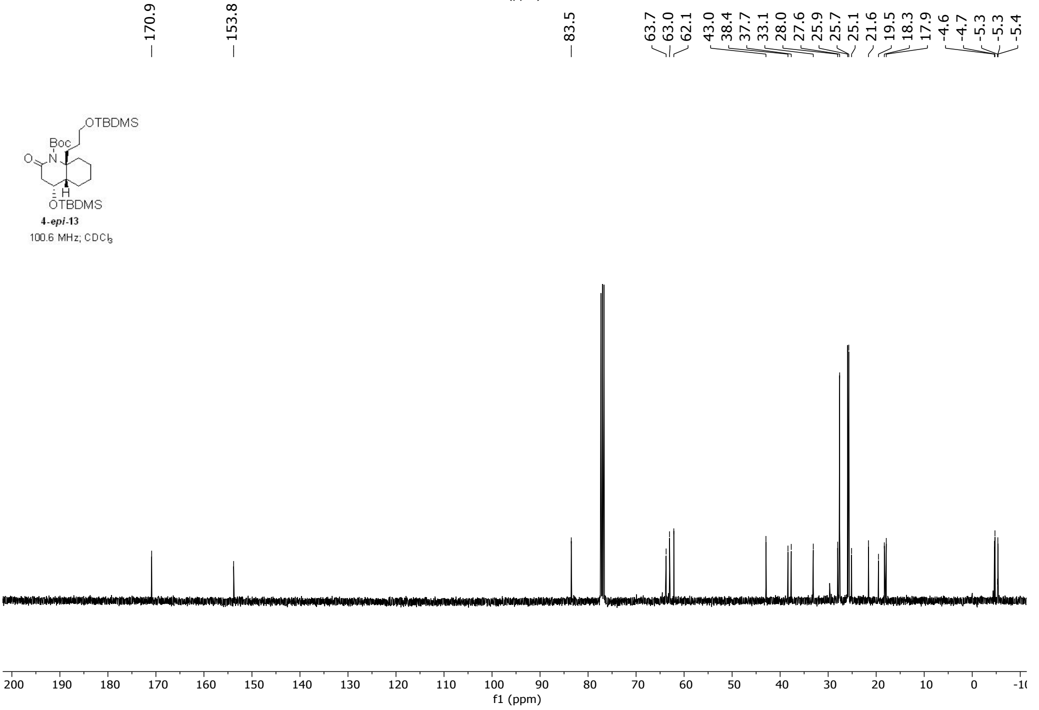
170.9
153.8
83.5
63.7
63.0
62.1
43.0
38.4
37.7
33.1
28.0
27.6
25.9
25.7
25.1
21.6
19.5
18.3
17.9
-4.6
-4.7
-5.3
-5.3
-5.4
OTBDMS
Boc
O
N
H
OTBDMS
4-epi-13
100.6 MHz; CDCl3
200
190
180
170
160
150
140
130
120
110
100
90
80
70
60
50
40
30
20
10
0
-1(
f1 (ppm)

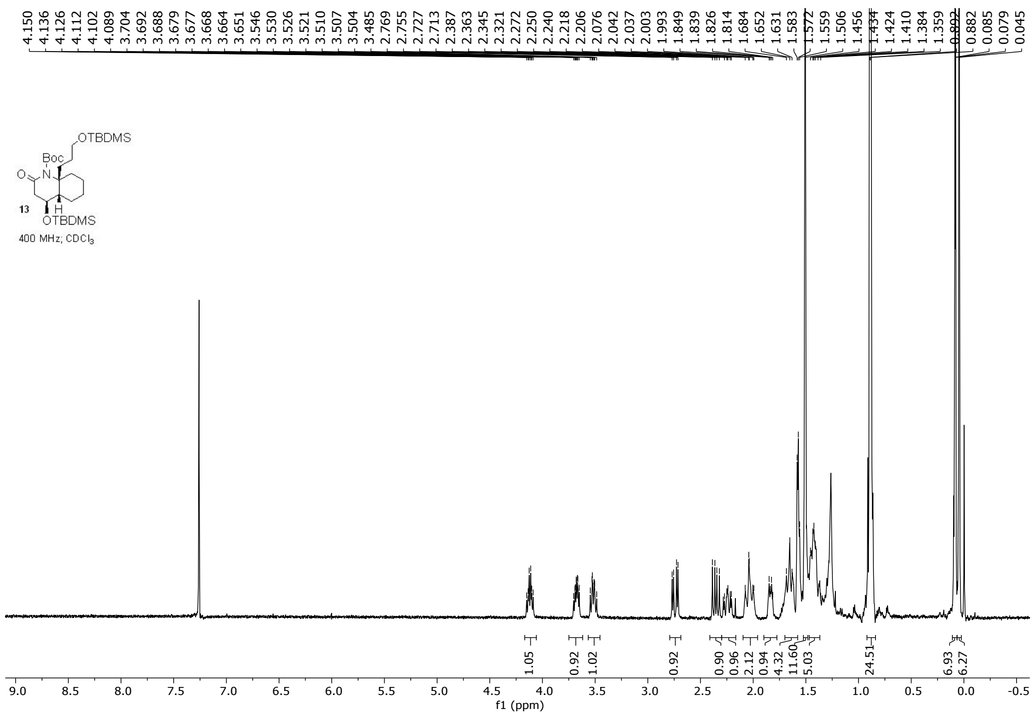

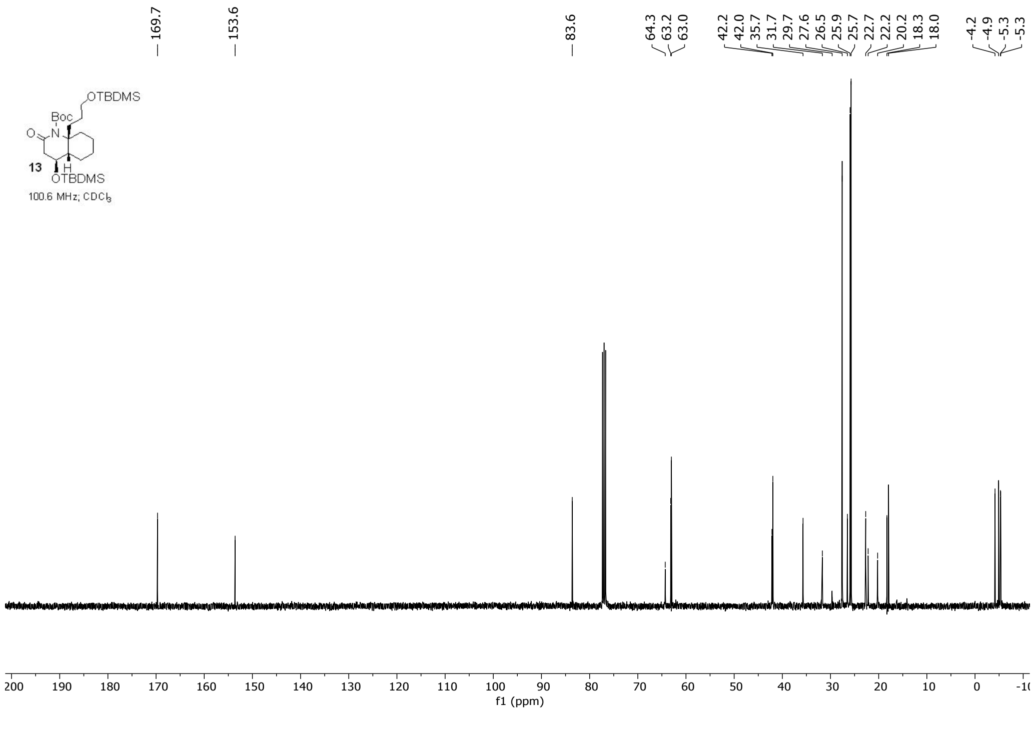

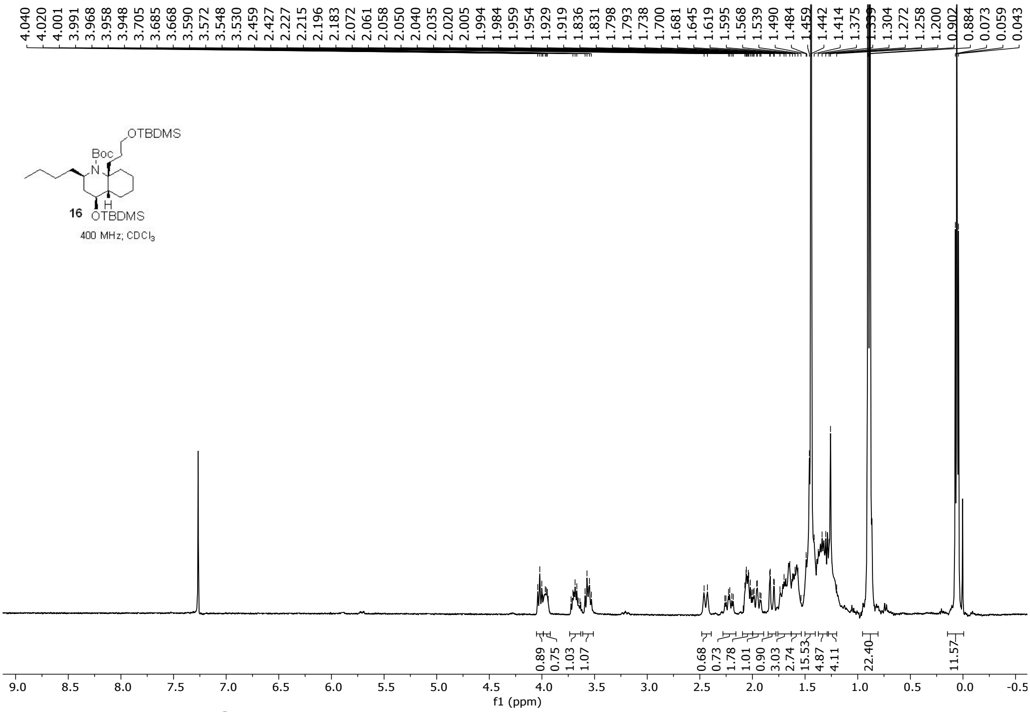
4.040
4.020
4.001
3.991
3.968
3.958
3.948
3.705
3.685
3.668
3.590
3.572
3.548
3.530
2.459
2.427
2.227
2.215
2.196
2.183
2.072
2.061
2.058
2.050
2.040
2.035
2.020
2.005
1.994
1.984
1.959
1.954
1.929
1.919
1.836
1.831
1.798
1.793
1.738
1.700
1.681
1.645
1.619
1.595
1.568
1.539
1.490
1.484
1.459
1.442
1.414
1.375
1.339
1.304
1.272
1.258
1.200
0.902
0.884
0.073
0.059
0.043
OTBDMS
Boc
N
H
16
OTBDMS
400 MHz; CDCl3
0.89
0.75
1.03
1.07
0.68
0.73
1.78
1.01
0.90
3.03
2.74
15.53
4.87
4.11
22.40
11.57
9.0
8.5
8.0
7.5
7.0
6.5
6.0
5.5
5.0
4.5
4.0
3.5
3.0
2.5
2.0
1.5
1.0
0.5
0.0
-0.5
f1 (ppm)

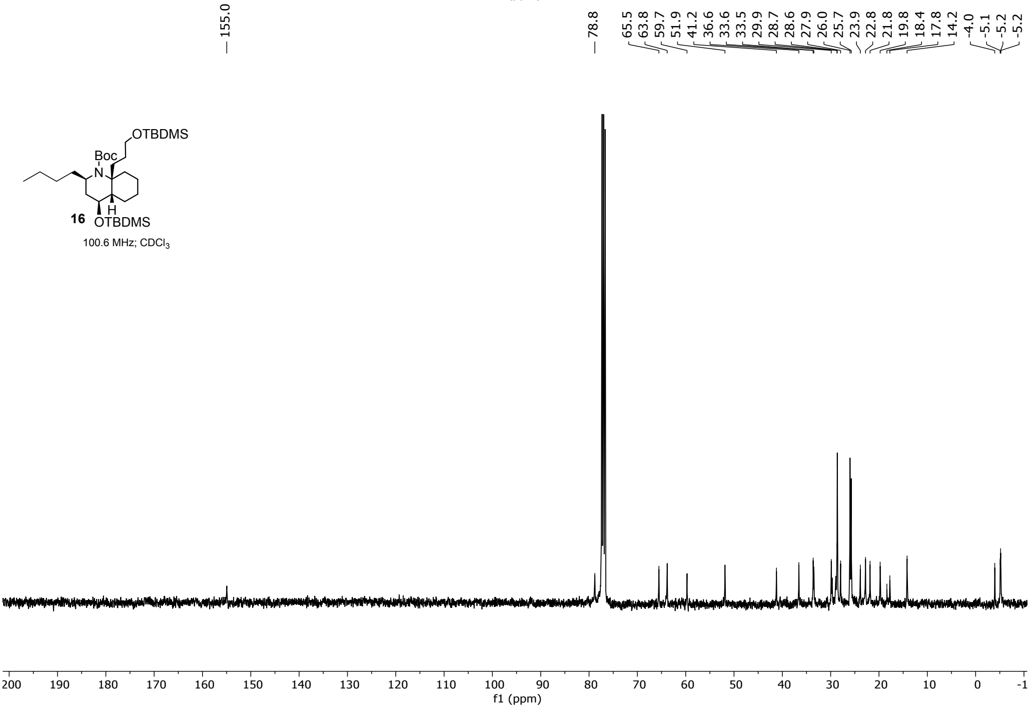
155.0
78.8
65.5
63.8
59.7
51.9
41.2
36.6
33.6
33.5
29.9
28.7
28.6
27.9
26.0
25.7
23.9
22.8
21.8
19.8
18.4
17.8
14.2
-4.0
-5.1
-5.2
-5.2
OTBDMS
Boc
N
H
16
OTBDMS
100.6 MHz; CDCl3
200
190
180
170
160
150
140
130
120
110
100
90
80
70
60
50
40
30
20
10
0
-1
f1 (ppm)

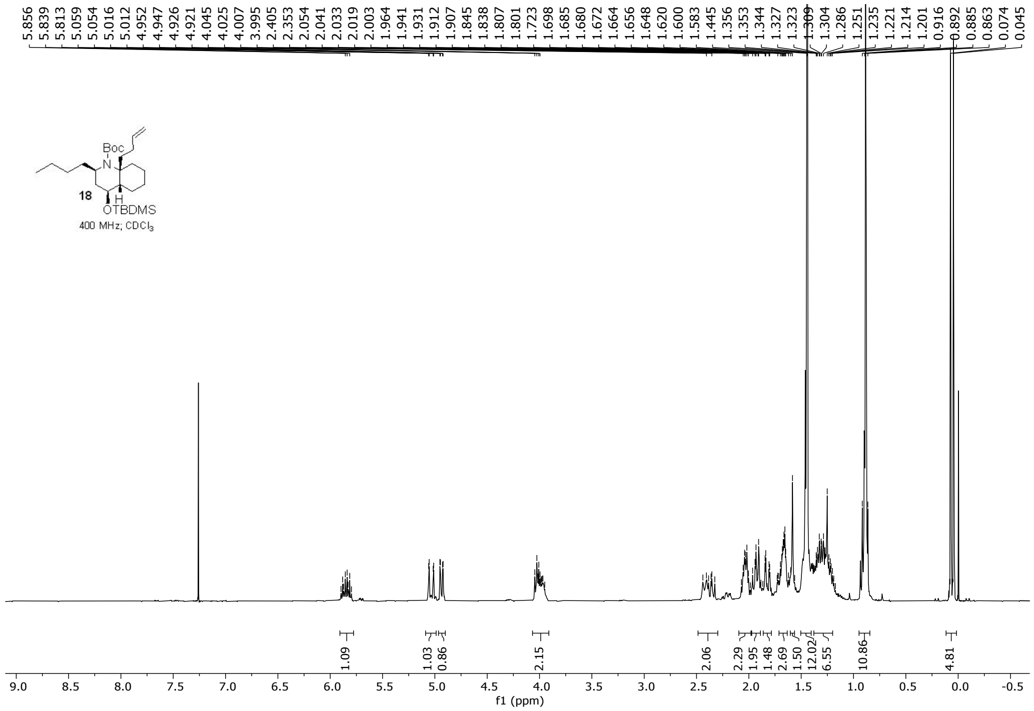
5.856 5.839 5.813 5.059 5.054 5.016 5.012 4.952 4.947 4.926 4.921 4.045 4.025 4.007 3.995 2.405 2.353 2.054 2.041 2.033 2.019 2.003 1.964 1.941 1.931 1.912 1.907 1.845 1.838 1.807 1.801 1.723 1.698 1.685 1.680 1.672 1.664 1.656 1.648 1.620 1.600 1.583 1.445 1.356 1.353 1.344 1.327 1.323 1.309 1.304 1.286 1.251 1.235 1.221 1.214 1.201 0.916 0.892 0.885 0.863 0.074 0.045
Boc
N
18
H
OTBDMS
400 MHz; CDCl3
1.09 1.03 0.86 2.15 2.06 2.29 1.95 1.48 2.69 1.50 12.02 6.55 10.86 4.81
9.0 8.5 8.0 7.5 7.0 6.5 6.0 5.5 5.0 4.5 4.0 3.5 3.0 2.5 2.0 1.5 1.0 0.5 0.0 -0.5
f1 (ppm)

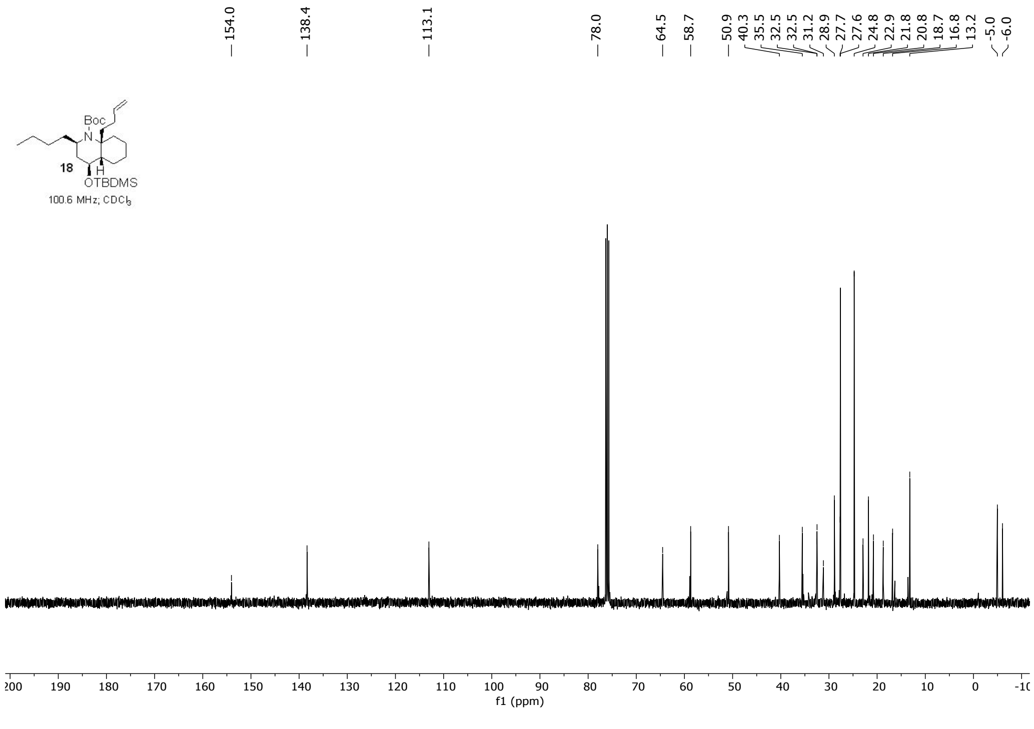
154.0 138.4 113.1 78.0 64.5 58.7 50.9 40.3 35.5 32.5 32.5 31.2 28.9 27.7 27.6 24.8 22.9 21.8 20.8 18.7 16.8 13.2 -5.0 -6.0
Boc
N
18
H
OTBDMS
100.6 MHz; CDCl3
200 190 180 170 160 150 140 130 120 110 100 90 80 70 60 50 40 30 20 10 0 -10
f1 (ppm)

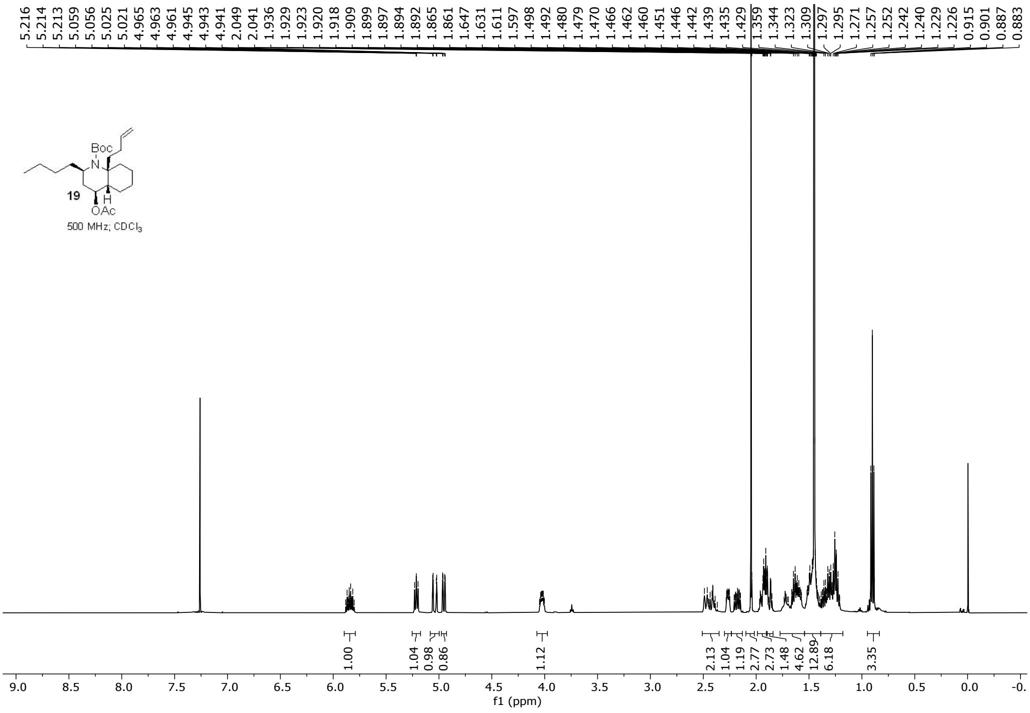
5.216 5.214 5.213 5.059 5.056 5.025 5.021 4.965 4.963 4.961 4.945 4.943 4.941 2.049 2.041 1.936 1.929 1.923 1.920 1.918 1.909 1.899 1.897 1.894 1.892 1.865 1.861 1.647 1.631 1.611 1.597 1.498 1.492 1.480 1.479 1.470 1.466 1.462 1.460 1.451 1.446 1.442 1.439 1.435 1.429 1.359 1.344 1.323 1.309 1.297 1.295 1.271 1.257 1.252 1.242 1.240 1.229 1.226 0.915 0.901 0.887 0.883
Boc
N
19
H
OAc
500 MHz; CDCl3
1.00 1.04 0.98 0.86 1.12 2.13 1.04 1.19 2.77 2.73 1.48 4.62 12.89 6.18 3.35
9.0 8.5 8.0 7.5 7.0 6.5 6.0 5.5 5.0 4.5 4.0 3.5 3.0 2.5 2.0 1.5 1.0 0.5 0.0
f1 (ppm)

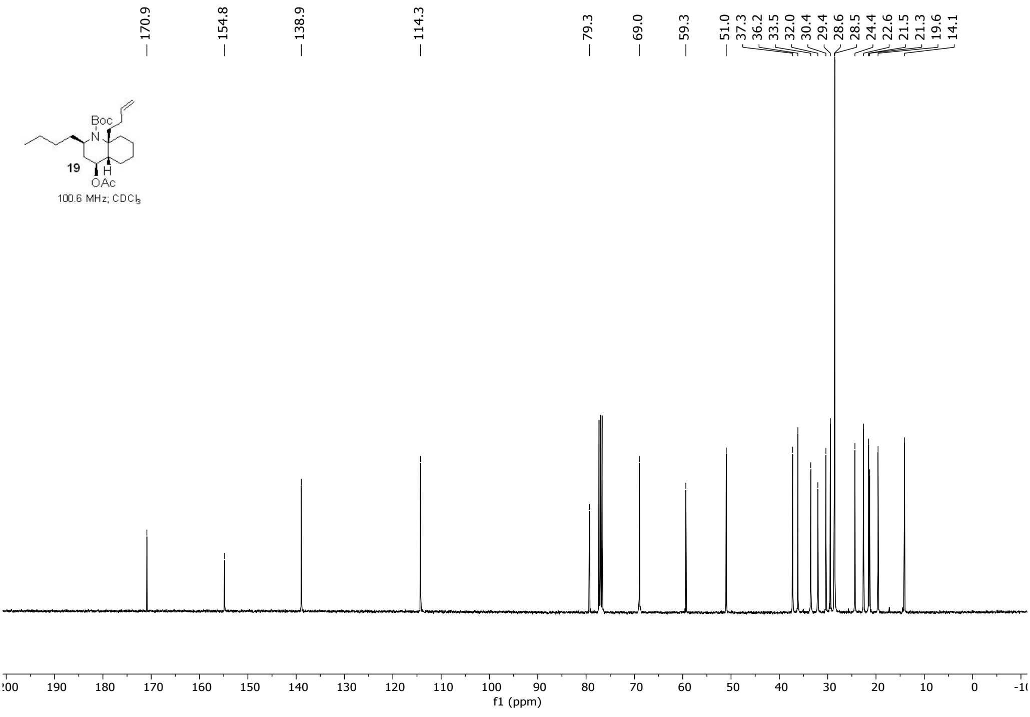
170.9 154.8 138.9 114.3 79.3 69.0 59.3 51.0 37.3 36.2 33.5 32.0 30.4 29.4 28.6 28.5 24.4 22.6 21.5 21.3 19.6 14.1
Boc
N
19
H
OAc
100.6 MHz; CDCl3
190 180 170 160 150 140 130 120 110 100 90 80 70 60 50 40 30 20 10 0
f1 (ppm)

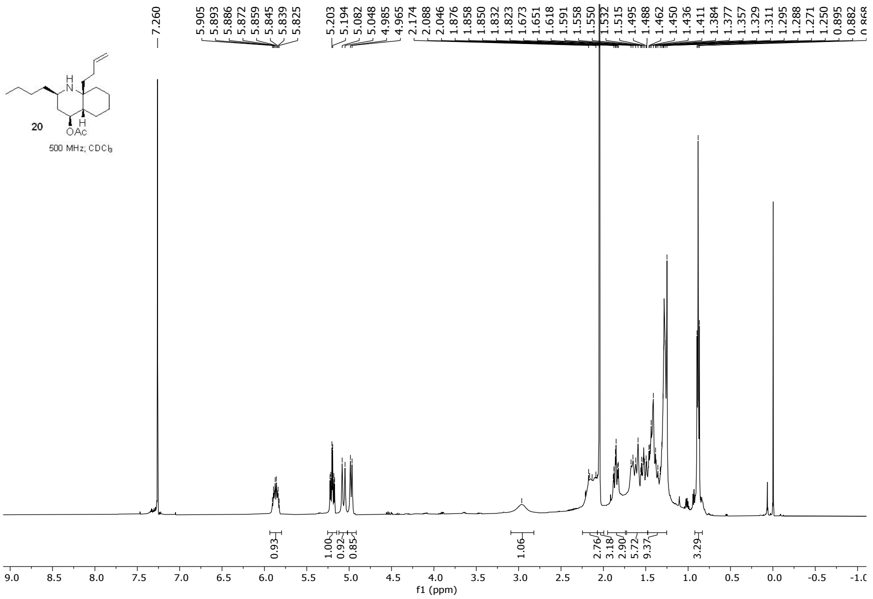
20
500 MHz; CDCl3
7.260
5.905 5.893 5.886 5.872 5.859 5.845 5.839 5.825
5.203 5.194 5.082 5.048 4.985 4.965
2.174 2.088 2.046 1.876 1.858 1.850 1.832 1.823 1.673 1.651 1.618 1.591 1.558 1.550 1.532 1.515 1.495 1.488 1.462 1.450 1.436 1.411 1.384 1.377 1.357 1.329 1.311 1.295 1.288 1.271 1.250 0.895 0.882
0.93 1.00 0.92 0.85 1.06 2.76 3.18 2.90 5.72 9.37 3.29
f1 (ppm)

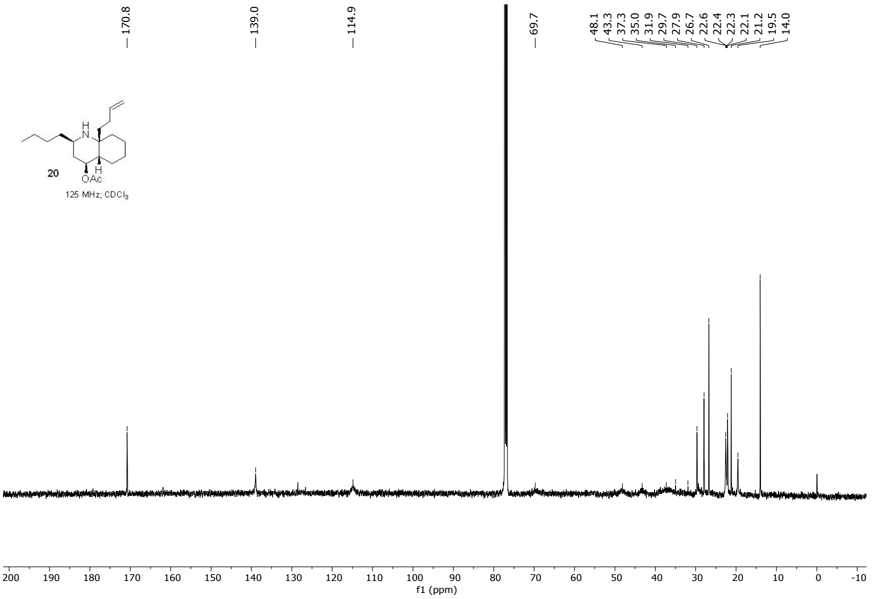
20
125 MHz; CDCl3
170.8
139.0
114.9
69.7
48.1 43.3 37.3 35.0 31.9 29.7 27.9 26.7 22.6 22.4 22.3 22.1 21.2 19.5 14.0
f1 (ppm)

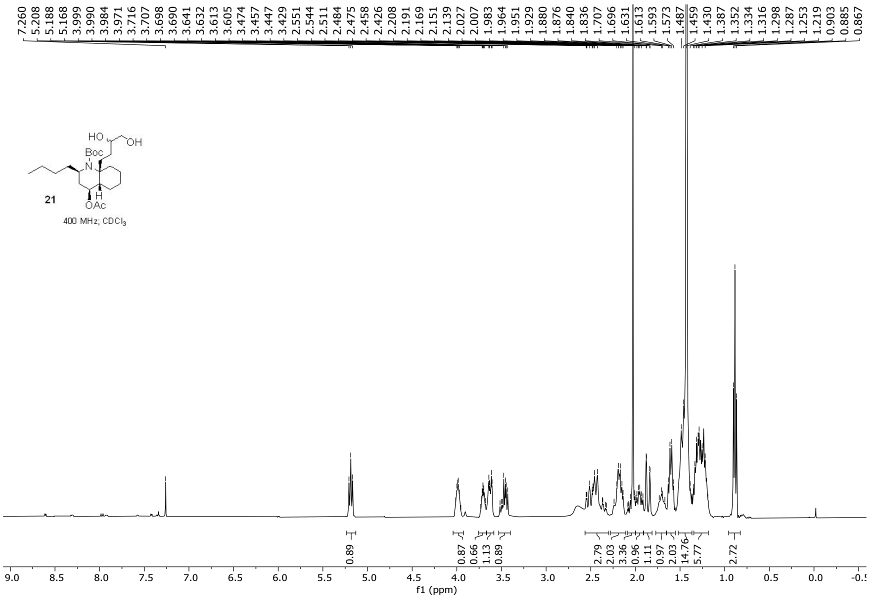
7.260 5.208 5.188 5.168 3.999 3.990 3.984 3.971 3.716 3.707 3.698 3.690 3.641 3.632 3.613 3.605 3.474 3.457 3.447 3.429 2.551 2.544 2.511 2.484 2.475 2.458 2.426 2.208 2.191 2.169 2.151 2.139 2.027 2.007 1.983 1.964 1.951 1.929 1.880 1.876 1.840 1.836 1.707 1.696 1.631 1.613 1.593 1.573 1.487 1.459 1.430 1.387 1.352 1.334 1.316 1.298 1.287 1.253 1.219 0.903 0.885 0.867
HO
OH
Boc
N
H
OAc
21
400 MHz; CDCl3
0.89 0.87 0.66 1.13 0.89 2.79 2.03 3.36 0.96 1.11 0.97 2.03 14.76 5.77 2.72
f1 (ppm)

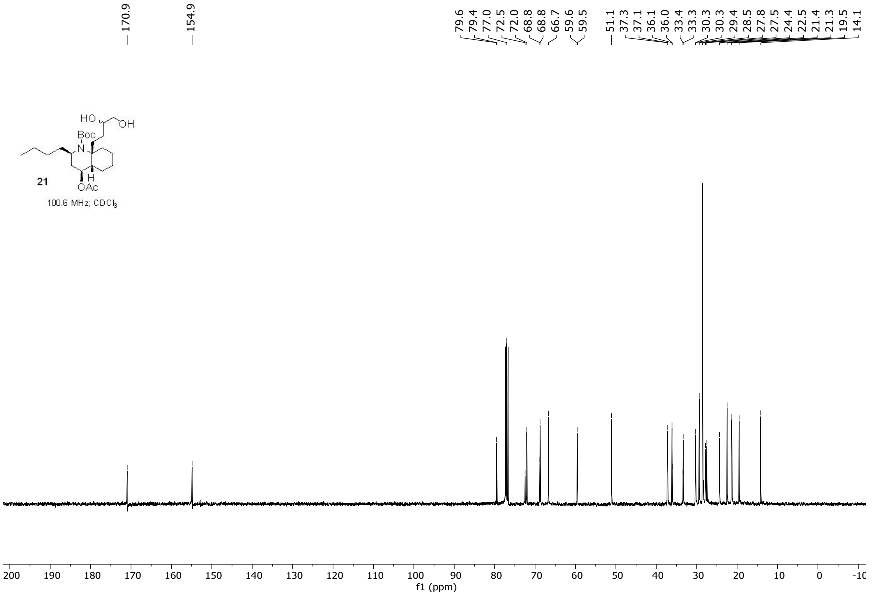
170.9 154.9 79.6 79.4 77.0 72.5 72.0 68.8 68.8 66.7 59.6 59.5 51.1 37.3 37.1 36.1 36.0 33.4 33.3 30.3 30.3 29.4 28.5 27.8 27.5 24.4 22.5 21.4 21.3 19.5 14.1
HO
OH
Boc
N
H
OAc
21
100.6 MHz; CDCl3
f1 (ppm)

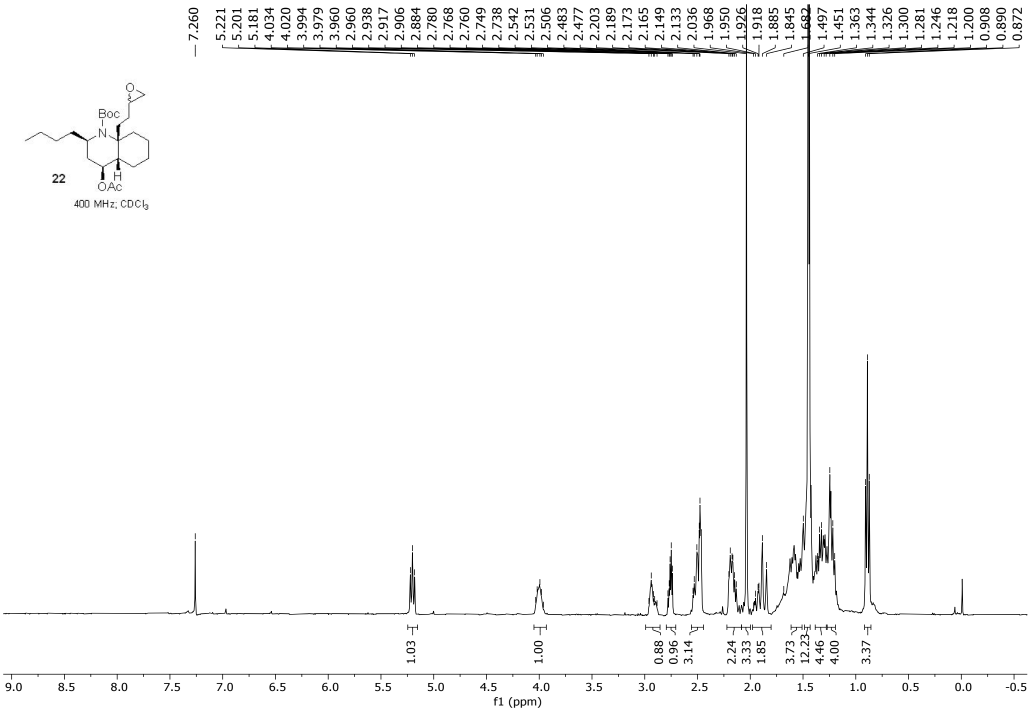
7.260
5.221
5.201
5.181
4.034
4.020
3.994
3.979
3.960
2.960
2.938
2.917
2.906
2.884
2.780
2.768
2.760
2.749
2.738
2.542
2.531
2.506
2.483
2.477
2.203
2.189
2.173
2.165
2.149
2.133
2.036
1.968
1.950
1.926
1.918
1.885
1.845
1.682
1.497
1.451
1.363
1.344
1.326
1.300
1.281
1.246
1.218
1.200
0.908
0.890
0.872
Boc
N
O
H
OAc
22
400 MHz; CDCl3
1.03
1.00
0.88
0.96
3.14
2.24
3.33
1.85
3.73
12.23
4.46
4.00
3.37
f1 (ppm)

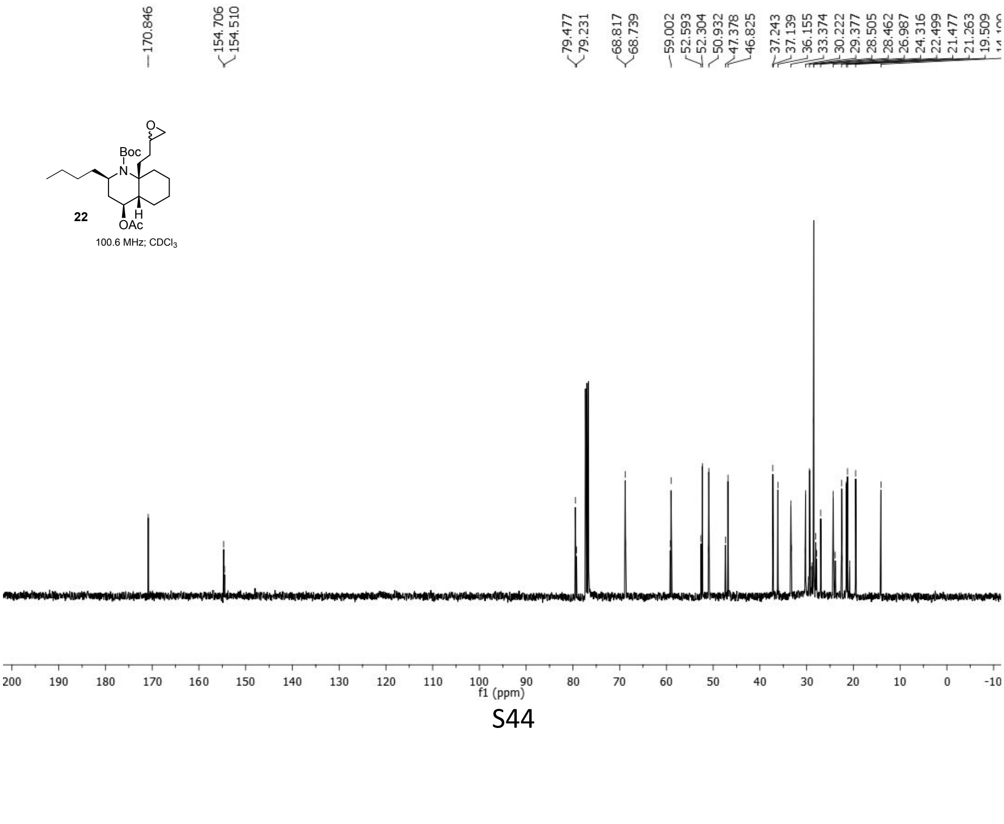
170.846
154.706
154.510
79.477
79.231
68.817
68.739
59.002
52.593
52.304
50.932
47.378
46.825
37.243
37.139
36.155
33.374
30.222
29.377
28.505
28.462
26.987
24.316
22.499
21.477
21.263
19.509
Boc
N
O
H
OAc
22
100.6 MHz; CDCl3
f1 (ppm)

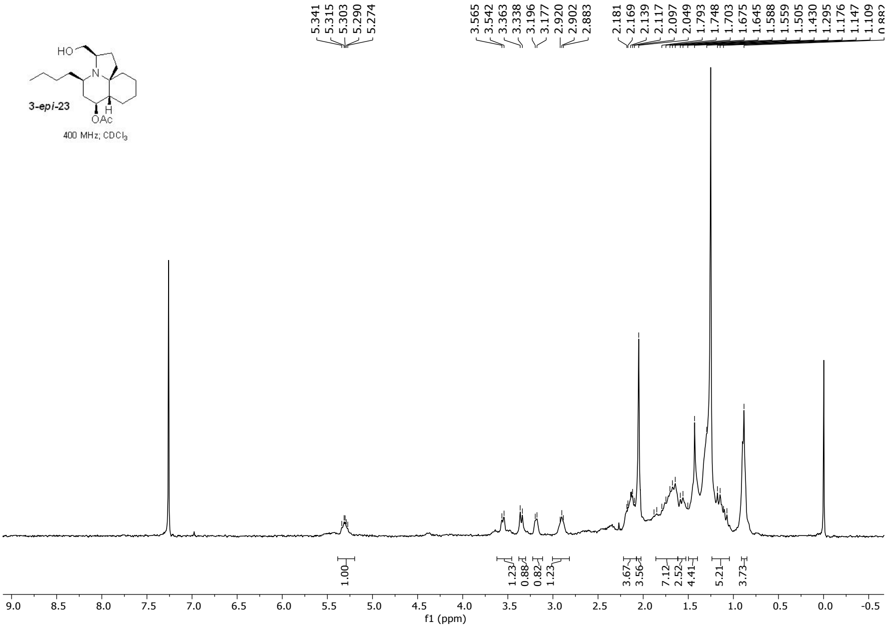
HO
N
3-epi-23
H
OAc
400 MHz, CDCl3
f1 (ppm)

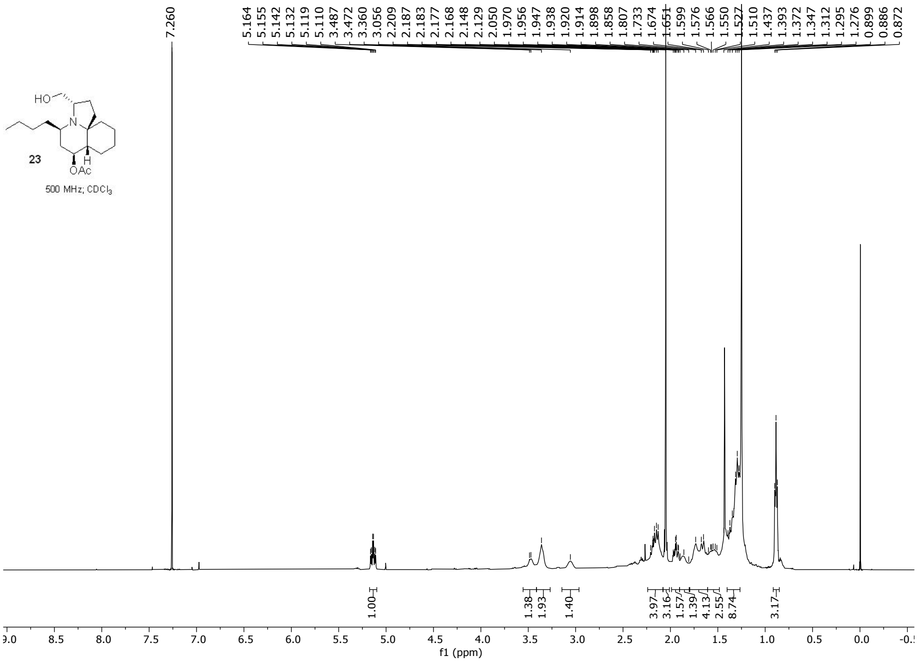

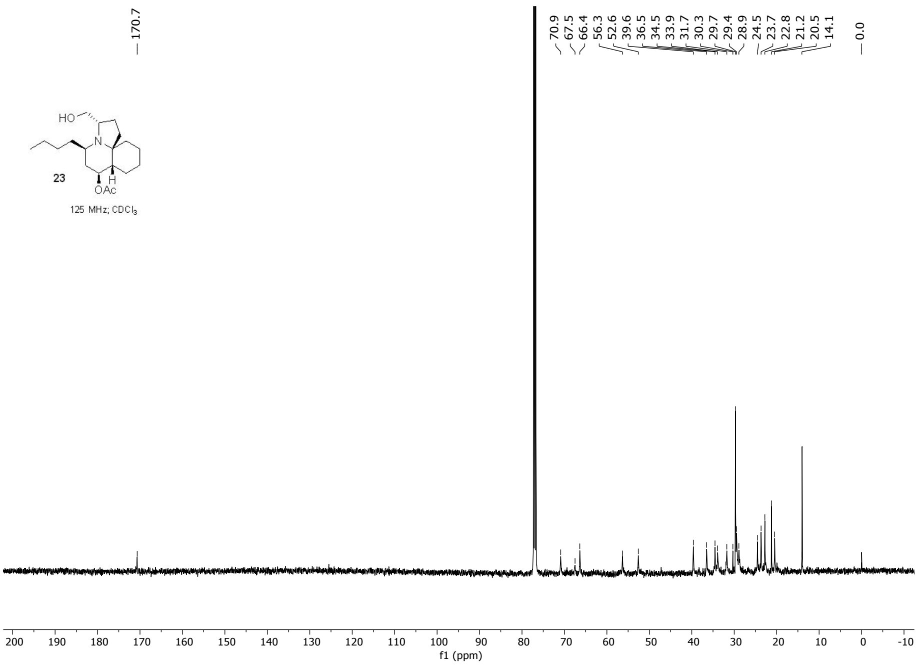

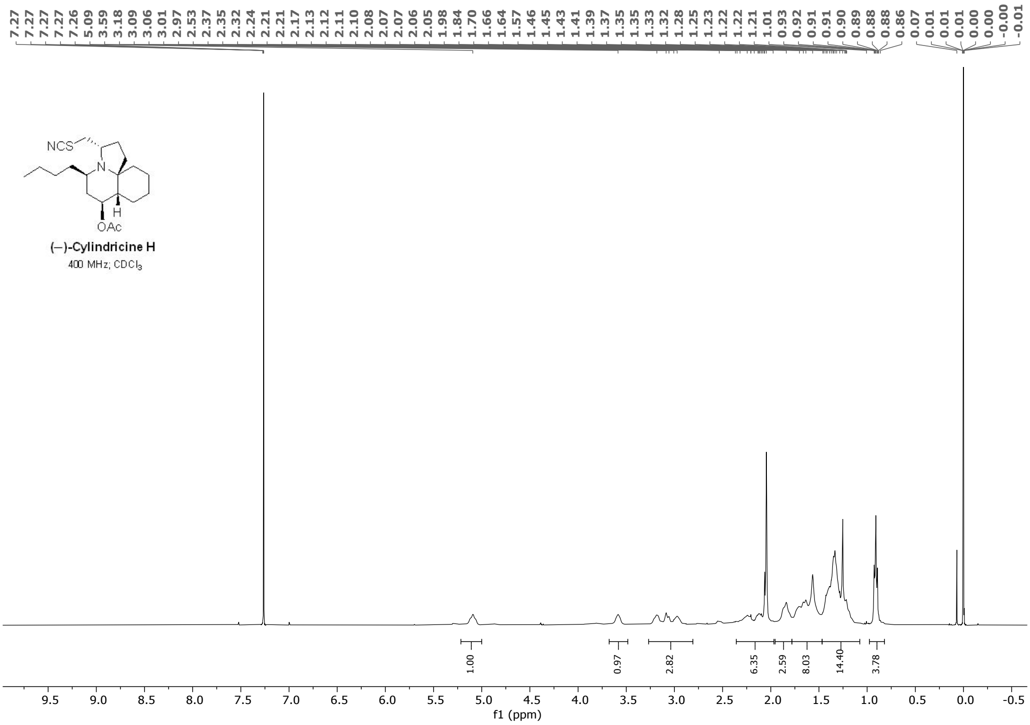
7.27 7.27 7.27 7.27 7.26 5.09 3.59 3.18 3.09 3.06 3.01 2.97 2.53 2.37 2.35 2.32 2.24 2.21 2.21 2.17 2.13 2.12 2.11 2.10 2.08 2.07 2.07 2.06 2.05 1.98 1.84 1.70 1.66 1.64 1.57 1.46 1.45 1.43 1.41 1.39 1.37 1.35 1.35 1.33 1.32 1.28 1.25 1.23 1.22 1.22 1.21 1.01 0.93 0.92 0.91 0.91 0.90 0.89 0.88 0.88 0.86 0.07 0.01 0.01 0.01 0.00 0.00 -0.00 -0.01
NCS
N
H
OAc
(–)-Cylindricine H
400 MHz; CDCl3
1.00 0.97 2.82 6.35 2.59 8.03 14.40 3.78
9.5 9.0 8.5 8.0 7.5 7.0 6.5 6.0 5.5 5.0 4.5 4.0 3.5 3.0 2.5 2.0 1.5 1.0 0.5 0.0 -0.5
f1 (ppm)

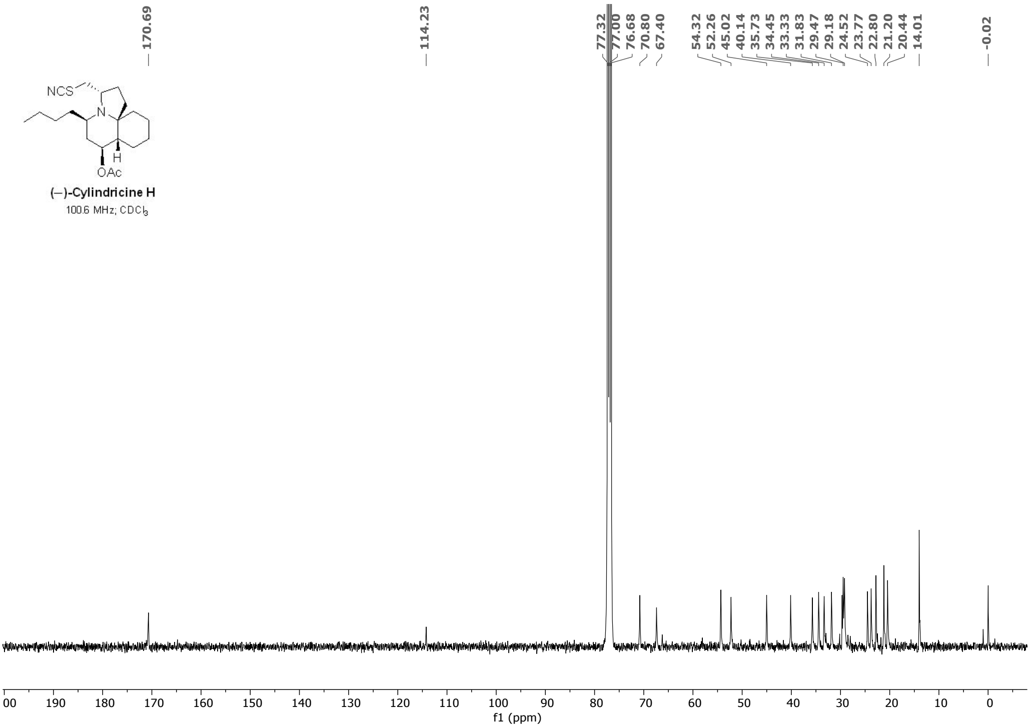
170.69 114.23 77.32 77.00 76.68 70.80 67.40 54.32 52.26 45.02 40.14 35.73 34.45 33.33 31.83 29.47 29.18 24.52 23.77 22.80 21.20 20.44 14.01 -0.02
NCS
N
H
OAc
(–)-Cylindricine H
100.6 MHz; CDCl3
00 190 180 170 160 150 140 130 120 110 100 90 80 70 60 50 40 30 20 10 0
f1 (ppm)
